# Highly Reversible Aqueous Anode‐Free Cadmium–Bromine Batteries

**DOI:** 10.1002/anie.1396057

**Published:** 2026-04-11

**Authors:** Xun Zhao, Yilong Zhu, Qianru Chen, Rujiao Ma, Junnan Hao, Shi‐Zhang Qiao

**Affiliations:** ^1^ School of Chemical Engineering Adelaide University Adelaide Australia

**Keywords:** aqueous anode‐free batteries, Br‐based cathode, Cd metal anode, high anode utilization, LiCl additive

## Abstract

Aqueous anode‐free zinc (Zn)‐based batteries promise high energy density; however, their reversibility and lifespan are hampered by dendritic growth, hydrogen evolution, and poor Zn utilization (<20%). Cadmium (Cd), a close analogue of Zn, presents a viable alternative. Here, we systematically compare both Zn and Cd anodes in aqueous media, showing that Cd features dendrite‐free deposition, suppressed side reactions, and stable cycling at anode utilization up to 75%. Benefiting from these advances, we demonstrate the first aqueous anode‐free cadmium–bromine (Cd–Br) battery. To boost Cd^2+^ plating kinetics in an anode‐free Cd–Br battery, LiCl is introduced into CdSO_4_ electrolyte to reconstruct the Cd^2+^ solvation shell, resulting in accelerated desolvation and deposition kinetics. Consequently, anode‐free Cd–Br coin cells achieve 87.6% capacity retention after 2000 cycles at 4 C, significantly superior to the anode‐free Zn–Br coin cells with 12.4% retention after only 50 cycles and outperforming other reported aqueous anode‐free systems. Moreover, scaled‐up aqueous anode‐free Cd–Br pouch cells exhibit stable cycling over 1250 cycles with capacity retention of 83.8% and high energy density of 157 Wh kg^−1^, far exceeding that of state‐of‐the‐art Zn–Mn and Zn–V pouch cells. This work establishes anode‐free Cd–Br chemistry as a new paradigm in developing high‐energy and highly reversible aqueous batteries.

## Graphical abstract

Cadmium and zinc anodes in aqueous electrolytes are compared, revealing that cadmium deposits smoothly with suppressed parasitic reactions and high metal utilization. Anode‐free cadmium–bromine batteries are then developed, where LiCl additive tunes the Cd^2+^ solvation structure to accelerate Cd^2+^ desolvation and plating kinetics. The anode‐free pouch cells deliver long cycling life and high energy density.

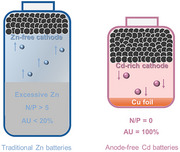

## 1 Introduction

Aqueous anode‐free (AF) battery concept has recently emerged as a compelling strategy to combine inherent safety advantages with boosted energy density [1, 2, 3, 4, 5, 6]. Aqueous AF zinc (Zn)‐based batteries operate without excess Zn metal anodes and rely on plating/stripping of Zn, thereby maximizing anode utilization (AU) and offering a simplified cell design with potentially higher energy density [7, 8, 9, 10, 11, 12]. However, the Zn anode is intrinsically unstable, undergoing spontaneous corrosion and hydrogen evolution reaction (HER) in mildly acidic electrolytes at both rest and operation conditions [13, 14, 15, 16, 17, 18, 19, 20, 21, 22, 23, 24]. Moreover, Zn dendrite formation leads to the short circuit and rapid failure of cells, especially under high currents [25, 26, 27, 28]. Consequently, Zn‐based batteries typically require flooded electrolytes and an excess Zn supply (<20% of Zn utilization), especially with high‐mass loading cathodes [29, 30, 31, 32], which severely reduces the overall energy density and limits the viability of aqueous AF Zn‐based batteries.

**FIGURE 1:**
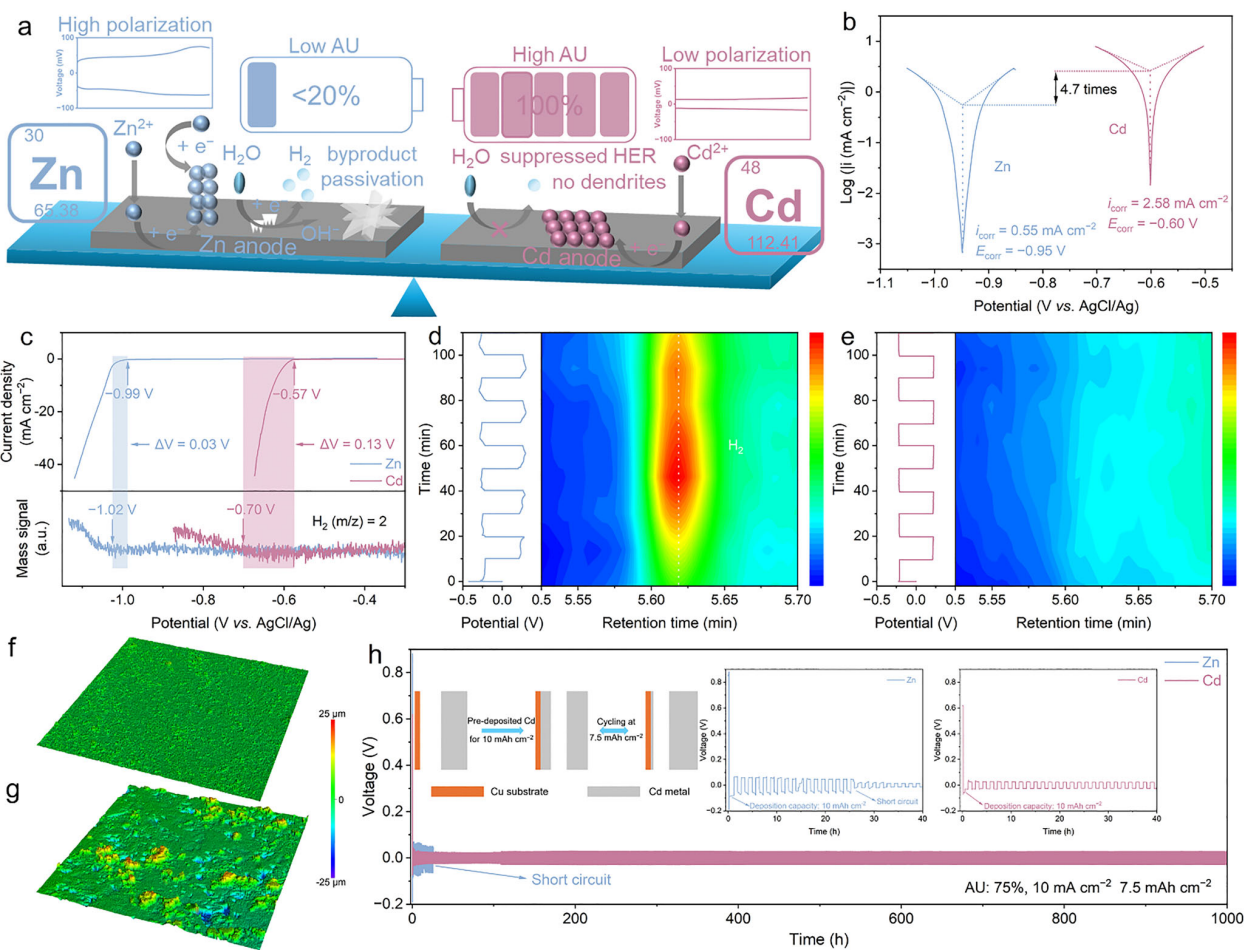
(a) Comparison of Zn and Cd metal anodes. (b) Tafel curves of Zn and Cd anodes. (c) LSV curves and in situ DEMS during Zn and Cd plating. In situ EC‐GC profiles of symmetric (d) Zn and (e) Cd batteries at 2 mA cm^−2^. 3D laser confocal images of (f) Cd and (g) Zn foils in symmetric batteries. (h) Cyclic stability of Zn–Zn and Cd–Cd half cells at a high AU of 75% and current density of 10 mA cm^−2^.

Cadmium (Cd), a structural analogue of Zn, provides distinctive electrochemical features that make it highly attractive for aqueous AF batteries [33]. Thermodynamically, Cd exhibits a less negative potential (−0.40 V vs. standard hydrogen electrode) than Zn, which reduces the driving force for parasitic H_2_ evolution and corrosion. Kinetically, the volcano‐type relationship between metal–hydrogen bonding strength and HER exchange current density places Cd on the low‐activity flank (Figure S1) [34, 35, 36], consistent with its lower HER activity and higher HER overpotential compared with Zn. Benefiting from the advances in both thermodynamics and kinetics, HER and by‐products formation, such as CdOHCl and Cd(OH)_2_, can be effectively suppressed. In addition, Cd anode exhibits lower polarization, dendrite‐free plating morphology, and higher AU than Zn, contributing to high reversibility in aqueous media (Figure 1a). Nevertheless, despite these favorable attributes, Cd metal anodes have attracted limited attention due to sluggish desolvation and Cd^2+^ deposition kinetics [37, 38]. Moreover, the AF Cd‐based batteries have not been demonstrated yet.

In this study, we systematically study the thermodynamic stability and electrochemical behavior of Zn and Cd metal anodes. Cd exhibits a significantly larger potential difference between the onset of metal deposition and HER (0.13 V) than Zn (0.03 V), suggesting a substantially reduced proclivity for HER. This suppression is further confirmed by in situ electrochemical–gas chromatography (EC–GC). Owing to the mitigated HER and dendrite‐free deposition, Cd–Cd half cells can operate stably for over 1000 h at a high AU of 75%, approximately 40 times longer than the Zn counterparts. Additionally, Cd anode displays a lower polarization (20 vs. 72 mV at 10 mA cm^−2^) and higher exchange current density (2.58 vs. 0.55 mA cm^−2^) compared to Zn anode, indicating superior electrochemical performance. Building on these advantages, we report the first AF cadmium–bromine (Cd–Br) battery, establishing a new aqueous battery chemistry. However, the sluggish Cd^2+^ deposition kinetics in 3 M CdSO_4_ (CSO) cause fluctuating Coulombic efficiency (CE) after a few cycles and eventual battery failure. To overcome this, we reconstruct the Cd^2+^ solvation shell by adding LiCl to CSO (denoted as CSO‐*x*LiCl, *x* = 1, 2, and 3), which enhances the plating/stripping reversibility. Under these conditions, an AF Cd–Br coin cell with a high mass loading of 10.6 mg cm^−2^ delivers an average CE of 99.13% and retains 87.6% capacity retention after 2000 cycles at 4 C, significantly superior to AF Zn–Br coin cells (12.4% retention after only 50 cycles, 283 times faster per‐cycle capacity loss) and other reported AF battery systems. Furthermore, the scaled AF Cd–Br pouch cells deliver stable operation over 1250 cycles with capacity retention of 83.8% and energy density of 157 Wh kg^−1^, surpassing state‐of‐the‐art Zn–Mn and Zn–V pouch systems. These results establish a viable pathway for developing high‐performance aqueous AF batteries.

## 2 Results and Discussion

### 2.1 Comparison of Zn and Cd Metal Anodes

Figure 1a presents a schematic illustration comparing Zn and Cd metal anodes regarding HER, byproduct passivation, AU, dendrite formation, and polarization. In aqueous Zn battery design, the Zn foil anode suffers from severe HER, byproduct formation, and dendritic growth, leading to higher polarization and low AU (generally less than 20%). In comparison, the Cd anode demonstrates lower polarization, suppressed HER, and dendrite‐free morphology, which enable the high reversibility and utilization ratio of Cd anodes and become a potential alternative to the Zn anode. The Tafel plots for Zn and Cd metal anodes in Figure 1b reveal considerable differences in corrosion current densities and potentials. Specifically, the exchange current density for Cd (2.58 mA cm^−2^) is approximately 4.7 times higher than for Zn (0.55 mA cm^−2^), indicating an easier reduction deposition process for Cd. Moreover, the corrosion potential of −0.60 V (vs. AgCl/Ag) for Cd is less negative than that of −0.95 V for Zn. Because HER is strongly coupled with Zn deposition in aqueous electrolytes, in situ differential electrochemical mass spectrometry (DEMS) enables direct distinction between the onset of HER and metal deposition by monitoring evolved gas in real time. Linear sweep voltammetry (LSV) combined with DEMS, illustrated in Figure 1c, further highlights different HER behaviors of Zn and Cd metal anodes [39, 40]. In this setup, an Au‐coated PTFE membrane serves as the working electrode, allowing H_2_ generated during deposition to pass into the vacuum chamber for mass spectrometric detection while blocking liquid electrolyte (Figure S2). The potential gap between Zn deposition and HER for the Zn electrode is notably narrow, at only 0.03 V, whereas Cd exhibits a broader window of 0.13 V, reflecting significantly reduced HER activity. In situ EC–GC (device illustrated in Figure S3) monitoring during plating/stripping process of Zn (Figures 1d and S4a) and Cd (Figures 1e and S4b) provides additional evidence [41, 42]. Zn displays pronounced H_2_ evolution (∼5.65 min), whereas Cd shows negligible H_2_ evolution under identical conditions, underscoring effective HER suppression.

Galvanostatic charge–discharge (GCD) curves of symmetric Zn and Cd batteries at various current densities (Figure S5), demonstrate that Zn anodes exhibit significantly higher polarization of 72 mV at 10 mA cm^−2^ compared to Cd anode (20 mV). Furthermore, cyclic voltammetry (CV) analyses in symmetric Zn and Cd batteries highlight considerable polarization (32 mV at a scan rate of 0.2 mV s^−1^) for Zn (Figure S6), contrasting with nearly instantaneous response for Cd, showing the higher electrochemical activity of Cd. 3D laser confocal microscopy images of the Cd (Figure 1f) and Zn (Figure 1g) metal surfaces in symmetric batteries were collected after 100 cycles at 5 mA cm^−2^. Cd metal clearly presented a smooth, uniform morphology surface. In comparison, the Zn metal surface shows a significantly increased surface roughness. The electrochemical performance of symmetric Zn and Cd metal half cells was systematically compared in 3 M ZnSO_4_ (ZSO) and CSO electrolytes, respectively. Under identical conditions, Cd half cells exhibit markedly superior cyclic stability at various current densities (0.2, 0.5, and 1 mA cm^−2^), as demonstrated in Figure S7. Importantly, pre‐deposited Cd–Cd cells demonstrate exceptional cyclic stability even at a high current density of 10 mA cm^−2^ across various AUs of 25% (Figure S8a), 50% (Figure S8b), and 75% (Figure 1h) [43]. In sharp contrast to the rapid short‐circuiting observed in the pre‐deposited Zn–Zn cells, Cd–Cd cells maintain stable operation beyond 1000 h, highlighting the excellent reversibility and robustness of Cd^2+^ plating/stripping. It is also noteworthy that Zn electrodes with high AU typically exhibit cycling lifetimes of less than 50 h.

### 2.2 Electrolyte Design for AF Cd–Br Batteries

**FIGURE 2:**
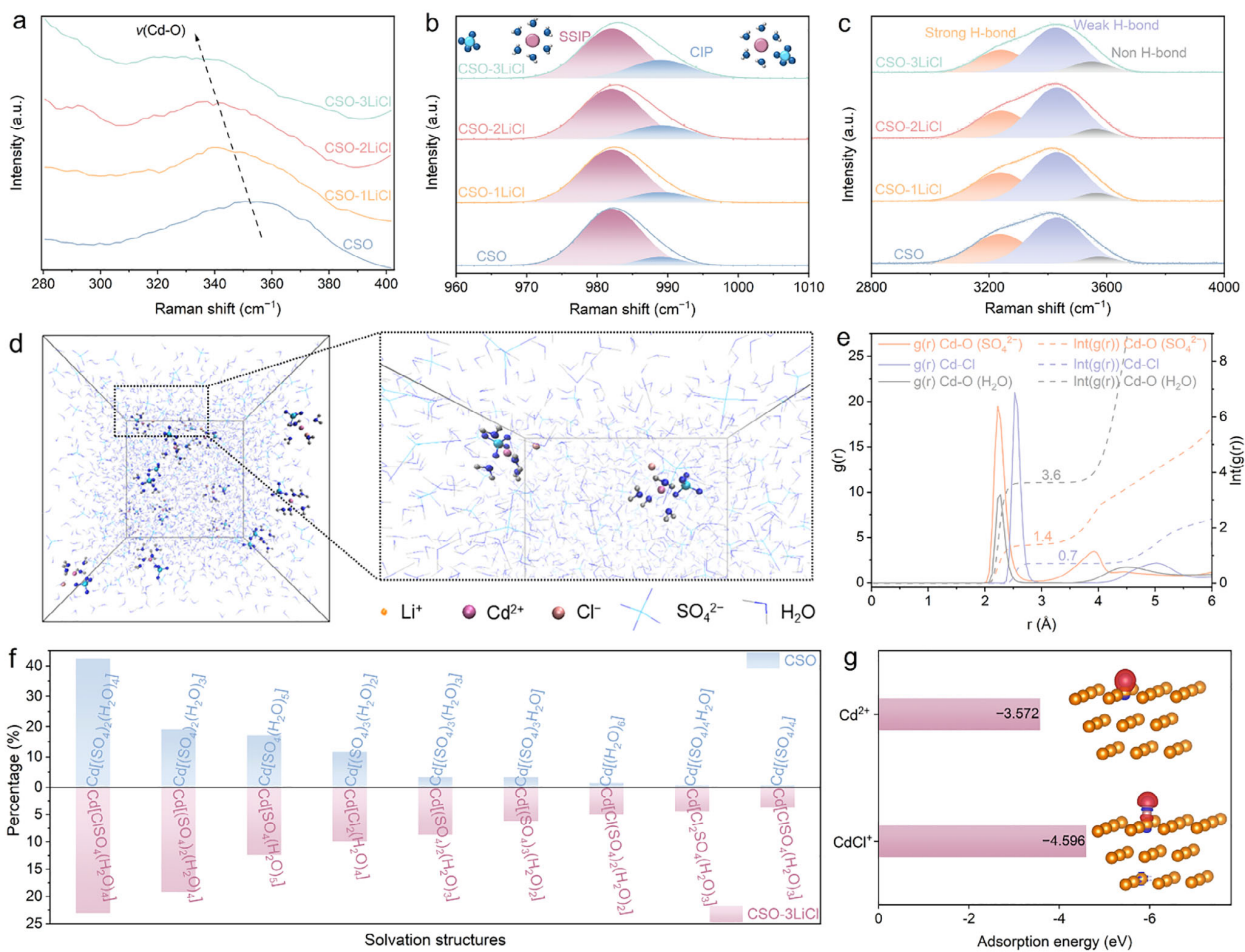
Raman spectra of CSO and CSO‐*x*LiCl showing (a) Cd–O stretching bands, (b) symmetric stretching vibration of SO_4_^2−^, and (c) O‐H stretching bands. (d) 3D snapshot of CSO‐3LiCl system from MD simulations and partial enlarged snapshot. (e) RDFs for Cd–O and Cd–Cl from MD simulations of CSO‐3LiCl. (f) Statistical distribution of Cd^2+^ solvation structures in CSO and CSO‐3LiCl with a coordination distance ≤ 3 Å. (g) Adsorption energy and charge density difference of Cd^2+^ and CdCl^+^ on Cu (111) facet.

Benefiting from the high AU without Cd metal anodes, AF Cd–Br batteries were assembled using deposited Cd on Cu foil as the active anode [44, 45]. However, in pristine CSO electrolyte, the poor reversibility of Cd plating on Cu foil under 100% utilization led to rapid cell failure, primarily due to sluggish Cd deposition kinetics (Figure S9). To address this issue, LiCl additive was introduced into the CSO electrolyte as a structure disruptor. Among different CSO‐*x*LiCl electrolytes, CSO‐3LiCl achieved a superior CE of 99.16% and exhibited stable operation exceeding 100 cycles (Figure S10), indicating the enhanced Cd deposition/dissolution kinetics by appropriate concentration of LiCl [46, 47, 48, 49]. To gain mechanistic insight into the effect of LiCl, a combination of spectroscopic characterizations and theoretical computations were performed. Raman spectroscopy was employed to investigate the solvation structure of Cd^2+^ and SO_4_^2−^ in CSO and CSO‐*x*LiCl. As shown in Figure 2a, increasing the LiCl concentration induces a gradual red shift and intensity decrease in the Cd–O Raman band [50], indicating significant alterations in the Cd^2+^ solvation structure and suggesting the possible participation of Cl^−^ in the coordination of Cd^2+^ by replacing SO_4_^2−^ and H_2_O. From the Raman signal of SO_4_^2−^ in Figure 2b, most Cd^2+^ ions in the CSO electrolyte exist as hexahydrate Cd[(H_2_O)_6_], corresponding to the solvation‐separated ion pairs (SSIP) at 982 cm^−1^, while only a small fraction of SO_4_^2−^ directly coordinates with Cd^2+^ to form contact ion pairs (CIP, Cd[SO_4_(H_2_O)_5_)]) at 989 cm^−1^ [51, 52]. With the gradual addition of LiCl, the Cd^2+^ solvation structure is altered, as Cl^−^ partially replaces SO_4_^2−^, while the remaining SO_4_^2−^ increases interaction with Li^+^. Consequently, the CIP signal is enhanced, accompanied by a decrease in SSIP intensity from CSO to CSO‐3LiCl. The O–H stretching region (Figure 2c) can be deconvoluted into five typical H‐bond species according to the proton donor–acceptor (D–A) model: DAA‐OH (3050 cm^−1^), DDAA‐OH (3200 cm^−1^), DA‐OH (3400 cm^−1^), DDA‐OH (3500 cm^−1^), and free‐OH (3650 cm^−1^) [53]. Among them, DAA‐OH and DDAA‐OH are generally assigned to strong H‐bond, DA‐OH and DDA‐OH correspond to weak H‐bond, and free‐OH corresponds to non H‐bond (Figure S11a) [54]. With increasing LiCl concentration, the fractions of weak and non H‐bonds increase, while that of strong H‐bonds species decreases (quantified in Figure S11b). These observations confirm LiCl as a structure disruptor of the H‐bond network, generating a more disordered yet electrochemically favorable solvation environment for Cd deposition. Further validation via Fourier transform infrared spectroscopy (FTIR, Figure S12a,b) demonstrated systematic blue shifts in symmetric SO_4_^2−^ stretching vibrations upon LiCl addition, supporting the inner structure change of Cd^2+^ and SO_4_^2−^. Similarly, blue shifts in O‐H stretching and bending vibrations (Figure S12c,d) indicated the weakened H‐bond network due to LiCl incorporation.

To further elucidate the effect of LiCl on the solvation structure of CSO, molecular dynamics (MD) simulations were performed for both CSO and CSO‐3LiCl systems (Tables S1 and S2). Representative MD snapshots in Figures 2d and S13 depict the spatial distribution of Cd^2+^, SO_4_^2−^, Li^+^, Cl^−^, and H_2_O molecules. Statistical analysis from MD simulation of the solvation environment reveals that, in the presence of LiCl, Cl^−^ and SO_4_^2−^ are incorporated into the first solvation shell of Cd^2+^, forming mixed‐anion coordination structures. Radial distribution functions (RDF, g(r) and lnt(g(r)), Figure S14) for the CSO system show the coordination numbers of 2.0 for Cd–O (SO_4_^2−^) and 3.8 for Cd–O (H_2_O). In contrast, the RDF of CSO‐3LiCl (Figure 2e) yields coordination numbers of 0.7 for Cd–Cl, 1.4 for Cd–O (SO_4_^2−^), and 3.6 for Cd–O (H_2_O). The strong Cd–Cl interaction is further confirmed by a pronounced peak at ∼2.525 Å, corresponding to an average of 0.7 Cl^−^ coordinated per Cd^2+^. These findings demonstrate that Cl^−^ partially replaces coordinated H_2_O molecules and SO_4_^2−^, resulting in a Cl^−^‐restructured solvation shell and weakened Cd^2+^‐H_2_O interaction [55]. Based on the simulation results, we quantified the specific Cd^2+^ solvation structures and their relative proportions in CSO and CSO‐3LiCl (Figure 2f; Tables S3 and S4). In CSO, the coordination environment is dominated by Cd[(SO_4_)_2_(H_2_O)_4_], Cd[(SO_4_)_2_(H_2_O)_3_], and Cd[SO_4_(H_2_O)_5_]. Upon adding LiCl, Cd[ClSO_4_(H_2_O)_4_] emerges as a major species in CSO‐3LiCl, together with Cd[(SO_4_)_2_(H_2_O)_4_] and Cd[SO_4_(H_2_O)_5_], indicating a clear reorganization of the primary solvation shell. Furthermore, theoretical calculations of adsorption energies on the Cu (111) facet yielded values of −3.572 eV for Cd^2+^ and −4.596 eV for CdCl^+^ (Figures 2g and S15), confirming enhanced interfacial binding facilitated by Cl^−^ coordination. Electrostatic potential analyses revealed significantly lower surface charge density for CdCl^+^ compared to Cd^2+^ (Figure S16), characterizing CdCl^+^ as a softer Lewis acid. Consequently, the superior adsorption behavior of CdCl^+^ can be attributed to favorable “soft–soft” interaction with the Cu substrate. From the charge density difference analysis in the inset of Figure 2g, red area represents the electron accumulation and blue area denotes electron depletion. These results further illustrate that bridging Cl^−^ ions between Cd species and the Cu surface promote adsorption.

### 2.3 Excellent Reversibility of Cd^2+^ Plating/Stripping in CSO‐3LiCl

**FIGURE 3:**
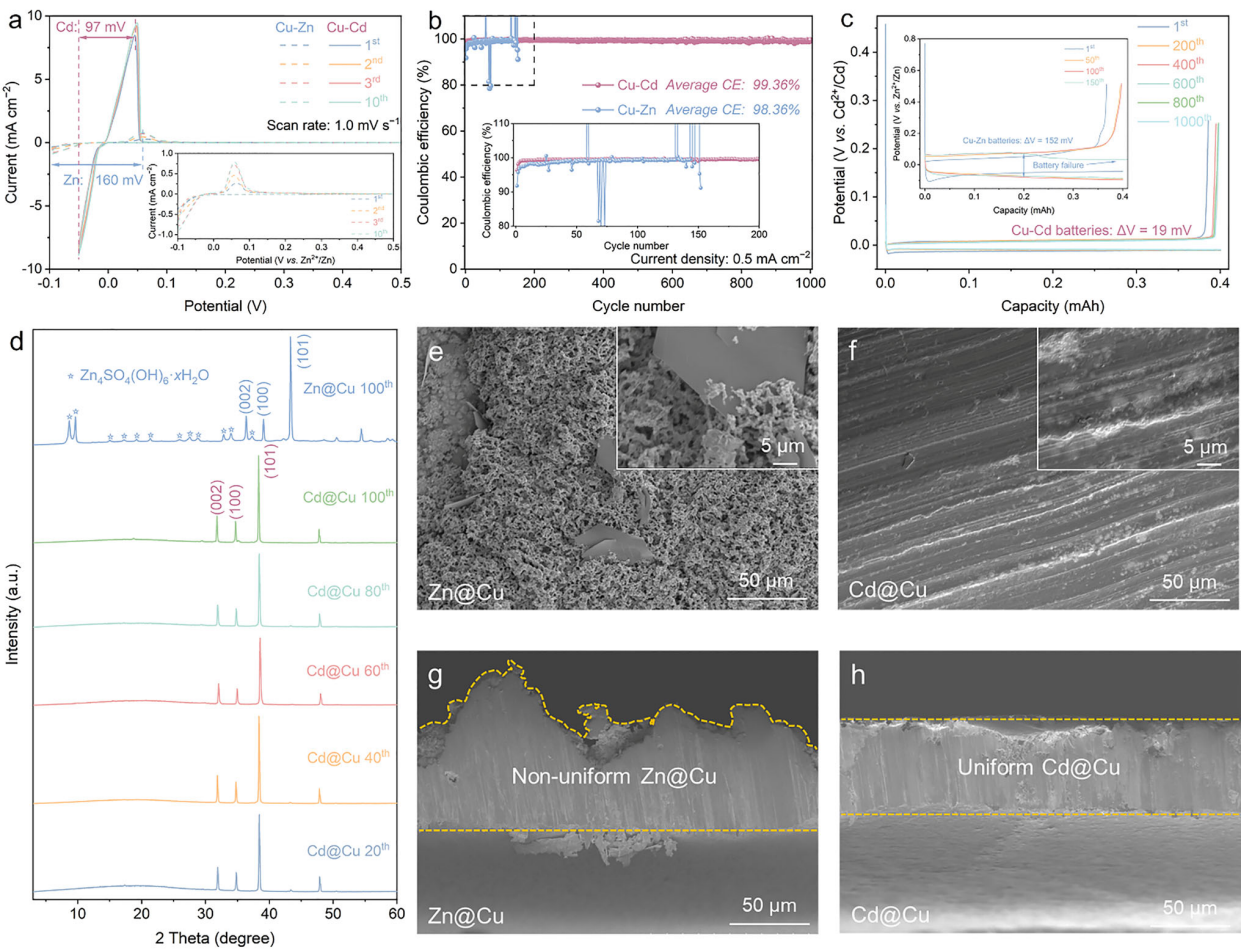
(a) CV curves of Cu–Cd (solid line) and Cu–Zn (dashed line) batteries at a scan rate of 1.0 mV s^−1^. (b) CE and (c) GCD curves of Cu–Cd and Cu–Zn batteries at 0.5 mA cm^−2^. (d) XRD patterns of plated Cd after different cycles with a deposition capacity of 5 mAh cm^−2^. Morphology of (e,f) surface and (g,h) cross section SEM images at a deposition capacity of 10 mAh cm^−2^.

CV curves obtained at a scan rate of 1.0 mV s^−1^ highlight substantial differences in nucleation behavior and reversibility between Cu–Cd (solid lines) and Cu–Zn (dashed lines) batteries, as shown in Figure 3a. Cu–Cd batteries exhibit significantly lower nucleation overpotential (∼97 mV), reflecting energetically favorable and efficient Cd deposition kinetics. In contrast, Cu–Zn batteries demonstrate a higher nucleation overpotential (∼160 mV, inset in Figure 3a), indicative of increased polarization and elevated energy barriers during Zn deposition. By contrast, for the AF Zn‐based systems, 3 M ZnSO_4_ + 3 m LiCl was not adopted as the benchmark electrolyte because both Cu–Zn and AF Zn–Br batteries exhibited markedly lower CE and faster failure than in pure ZSO (Figures S17 and S18), indicating aggravated parasitic reactions and interfacial instability rather than insufficient optimization. Therefore, ZSO was used for Zn‐based systems, whereas CSO‐3LiCl was used for Cd‐based cells. In Figure 3b, CE evaluations during extended cycling at 0.5 mA cm^−2^ indicate that Cu–Cd batteries maintain an impressive average CE of 99.36% over 1000 cycles. Conversely, Cu–Zn batteries exhibit a comparatively lower average CE of 98.36%, reflecting less reversible deposition/stripping processes attributed to parasitic side reactions, such as HER and Zn dendrite formation. GCD curves for Cu–Cd batteries (Figure 3c) consistently display stable and flat voltage plateaus throughout 1000 cycles, highlighting excellent cycling reversibility and minimal polarization increase. These results confirm the superior electrochemical stability and robustness of Cu–Cd systems. Additionally, Cu–Cd batteries demonstrate higher average CE values under different current density and areal capacity (0.2 mA cm^−2^/0.2 mAh cm^−2^ and 0.5 mA cm^−2^/0.5 mAh cm^−2^), as depicted in Figure S19. X‐ray diffraction (XRD) patterns (Figure 3d) of Cd and Zn deposited on Cu substrates after various cycles at a fixed deposition capacity of 5 mAh cm^−2^ further illustrate differences in structural stability and purity. The prominent diffraction peaks at 31.8°, 34.8°, and 38.4° corresponding to metallic Cd (002), (100), and (101), maintain consistent intensity and sharpness over cycling, signifying highly crystalline and stable metallic Cd deposits without significant phase transformations or structural degradation. In sharp contrast, Zn electrode exhibits distinct peaks at 8.7° and 9.6° corresponding to zinc hydroxide sulfate (Zn_4_SO_4_(OH)_6_·*x*H_2_O) after 100 cycles, suggesting extensive passivation and electrolyte consumption.

Scanning electron microscopy (SEM) analysis of metal deposition morphology at a capacity of 10 mAh cm^−2^ reveals notable morphological disparities. Zn deposition manifests as mossy structures accompanied by sheet‐like byproducts (Figures 3e and S20), indicative of uneven deposition and substantial polarization. Conversely, Cd deposition exhibits uniform, dense, and compact morphologies without visible dendritic features, indicative of superior deposition uniformity and reduced polarization (Figure 3f and S21). Cross section SEM images (Figures 3g,h, and S22) further show the high‐quality deposition of Cd at different areal capacity of 5 and 10 mAh cm^−2^. At both capacities, the deposited Cd layers appear compact, dense, and firmly adhered to the Cu foil, emphasizing strong interfacial adhesion and uniform plating. In contrast, cross section SEM images of Zn deposition reveal irregular and zigzag morphologies, highlighting inferior deposition quality and stability.

### 2.4 Electrochemical Performance of AF Cd–Br Coin Cells

**FIGURE 4:**
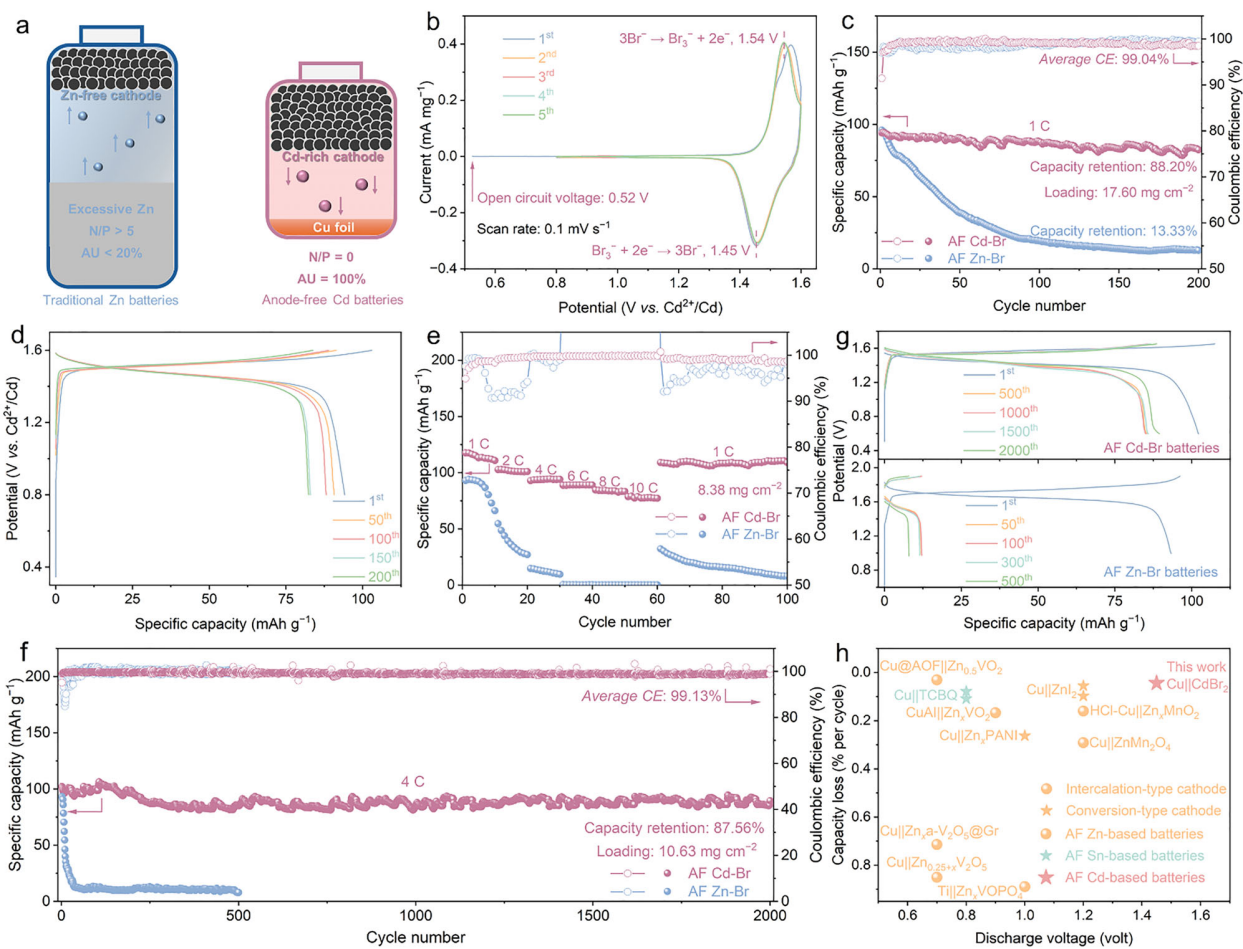
(a) Schematic illustration of traditional Zn batteries with low AU and AF Cd–Br batteries with high AU. (b) CV curves of AF Cd–Br batteries at 0.1 mV s^−1^. (c) Cyclic stability and (d) GCD curves of AF Cd–Br and Zn–Br batteries at 1 C. (e) Rate capability of AF Cd–Br and Zn–Br batteries. (f) Cyclic stability and (g) GCD curves of AF Cd–Br and Zn–Br batteries at 4 C. (h) Voltage and capacity loss comparison of AF Cd–Br batteries with other aqueous AF systems.

Figure 4a highlights significant performance disparities between traditional Zn‐based batteries and AF Cd‐based batteries. Traditional Zn–Br batteries typically suffer from a low AU of less than 20%, necessitating excessive Zn metal, which results in poor energy density and substantial volumetric inefficiencies. Conversely, AF Cd–Br batteries eliminate excess metal anode (N/P = 0), achieving 100% AU through direct Cd plating onto a Cu current collector. CV curves form the initial five cycles at a scan rate of 0.1 mV s^−1^ (Figure 4b) display two distinct and highly reversible redox peaks at 1.45 and 1.54 V (vs. Cd^2+^/Cd), corresponding to the Br_3_^−^/Br^−^ redox couple [56, 57]. The open circuit voltage of AF Cd–Br batteries is 0.52 V, notably less than the approximately 1.50 V observed for conventional Cd–Br batteries. In contrast, CV curves of AF Zn–Br batteries (Figure S23) show rapid current decay and increased polarization, indicating significant HER activity and byproduct accumulation. Figure 4c presents the superior cyclic stability of AF Cd–Br batteries with 88.2% capacity retention after 200 cycles at 1 C, coupled with a high mass loading of 17.6 mg cm^−2^, areal capacity of 2 mAh cm^−2^, and an average CE of 99.04%. Conversely, AF Zn–Br batteries exhibit rapid capacity decay and retain only 13.3% capacity, primarily due to irreversible Zn plating/stripping and severe HER. AF Cd–Br batteries (Figure 4d) demonstrate stable GCD curves at 1 C, in sharp contrast to AF Zn–Br batteries (Figure S24), which undergo significant capacity degradation within the initial 50 cycles.

Evaluation of rate capability demonstrates that AF Cd–Br batteries (Figures 4e and S25) maintain high specific capacities of 115.6 mAh g^−1^ at 1 C and 78.6 mAh g^−1^ at 10 C, showing minimal capacity loss even at a high mass loading of 8.38 mg cm^−2^. This reveals the superior kinetics and efficient ion transport in AF Cd–Br batteries. Conversely, AF Zn–Br batteries display significant capacity drops, poor retention, and a specific capacity of 50 mAh g^−1^ even at 2 C. The self‐discharge performance was evaluated by resting AF Cd–Br batteries for 12 h after charging (Figure S26). AF Cd–Br batteries exhibit a notably low self‐discharge rate, retaining 95.26% of their capacity, underscoring effective suppression of Br_3_^−^ shuttle effects and enhanced interfacial stability. By extending rest for up to 72 h, it shows a capacity retention of 79.59%, further confirming the low self‐discharge behavior. Long‐term cycling test at 4 C (Figure 4f,g) reveals that AF Cd–Br batteries maintain exceptional stability, with capacity retention of 87.56% after 2000 cycles and an impressive average CE of 99.13%, markedly outperforming all previously reported aqueous AF system in cycling stability (Figure 4h and Table S5). In contrast, AF Zn–Br batteries suffer severe capacity deterioration within the initial 50 cycles under identical conditions. Moreover, AF Cd–Br batteries retain 73.09% of their capacity after 5000 cycles at a higher rate of 8 C, while AF Zn–Br batteries experience rapid capacity decay within the initial 100 cycles (Figures S27 and S28).

### 2.5 Fabricating Large‐Format AF Cd–Br Pouch Cells

**FIGURE 5:**
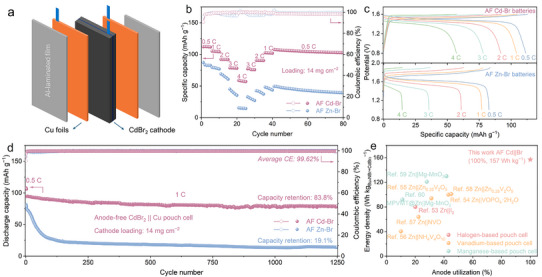
(a) Schematic illustration of AF Cd–Br pouch cells. (b) Rate capability and (c) GCD curves of AF Cd–Br and Zn–Br pouch cells. (d) Cyclic stability of AF Cd–Br and Zn–Br pouch cells at 1 C. (e) Energy density comparison of AF Cd–Br pouch cells with the reported Zn‐based pouch cells.

Figure 5a provides a schematic illustration of an AF Cd–Br pouch cell, comprising two Cu foils and a Br‐based cathode separated by an electrolyte‐soaked separator, all encapsulated within laminated aluminum pouch film. This compact architecture with high AU effectively eliminates excess metal anodes, enhancing overall energy density. The rate capability and GCD curves of AF Cd–Br and Zn–Br pouch cells are presented in Figure 5b,c. The AF Cd–Br pouch cell with a high mass loading of 14 mg cm^−2^ exhibits outstanding rate performance over a wide rate range, delivering capacities of 111, 104, 92, 78, and 57 mAh g^−1^ at 0.5 C, 1 C, 2 C, 3 C, and 4 C, respectively. Even under fast charge–discharge conditions, the AF Cd–Br system maintains high specific capacities with superior reversibility. By contrast, AF Zn–Br pouch cells deliver 87.2, 79.6, 69.1, 45.1, and 15.5 mAh g^−1^ at the same rates but experience significant capacity decay at elevated rates, reflecting the poor rate capability of the AF Zn–Br system arising from uneven Zn deposition and parasitic side reactions. Figures 5d and S29 depict the long‐term cycling performance and corresponding GCD curves of AF Cd–Br and Zn–Br pouch cells under 1 C. The AF Cd–Br pouch cell demonstrates remarkable cycling stability, achieving 83.8% capacity retention over 1250 cycles and an average CE of 99.62%. Conversely, AF Zn–Br pouch cells exhibit pronounced capacity degradation, retaining only 19.1% of their initial capacity after the same cycles, underscoring the markedly superior structural and interfacial stability of the AF Cd–Br system.

The Ragone plot in Figure 5e compares energy density versus AU for the AF Cd–Br pouch cell developed in this work with a range of reported aqueous Zn‐based pouch cell systems, including halogen‐based [58], vanadium‐based [59, 60, 61, 62, 63], and manganese‐based [64, 65] chemistries. For instance, Ji and coworkers demonstrated a ZnCl_2_‐based highly concentrated electrolyte in a Zn||VOPO_4_·2H_2_O pouch cell, achieving an AU of 43% and an energy density of 100 Wh kg^−1^ [59]. Similarly, Zhi's group reported a Zn(OTf)_2_/sulfolane/H_2_O reverse micelle electrolyte enabling an ampere‐hour scale Zn||Zn_0.25_V_2_O_5_·*n*H_2_O pouch cell with an energy density of 94 Wh kg^−1^ [60]. In comparison, the AF Cd–Br pouch cell achieves a markedly higher energy density of 157 Wh kg^−1^(calculated based on the mass of active materials in both cathode and anode), while simultaneously delivering 100% AU by eliminating excess Cd metal. This performance substantially surpasses most Zn‐based systems summarized in Table S6, which typically exhibit limited Zn utilization (<20%) and consequently much lower energy densities.

## 3 Conclusion

In this study, we report the first aqueous AF Cd–Br battery with excellent electrochemical performance, which significantly enriches the aqueous battery family. Initially, we systematically compare the behaviors of Zn and Cd metal anodes in aqueous battery systems, demonstrating that Cd exhibits lower polarization, smoother deposition morphology, reduced side reactions, and enhanced AU compared to the Zn anode. To boost Cd^2+^ deposition kinetics, CSO electrolyte is engineered by adding LiCl to reconstruct the Cd^2+^ solvation shell, thereby accelerating desolvation and Cd^2+^ plating kinetics. MD simulations further reveal that Cl^−^ partially substitutes H_2_O molecules and SO_4_^2−^ ions in the solvation shell of Cd^2+^. Specifically, AF Cd–Br coin cells achieve a remarkable capacity retention of 87.56% after 2000 cycles at 4 C with a high mass loading of 10.6 mg cm^−2^. At a higher rate of 8 C, the cell remains 73.09% of its capacity after 5000 cycles, outperforming other reported aqueous AF systems. In contrast, AF Zn–Br batteries experience rapid capacity degradation primarily due to parasitic reactions and uneven Zn dendrite growth. Moreover, the assembled large‐sized AF Cd–Br pouch cell demonstrates outstanding stability, maintaining stable cycle performance over 1250 cycles and delivering a high energy density of 157 Wh kg^−1^ based on the active materials. This finding comprehensively evaluates Zn and Cd metal anodes and introduces an innovative AF Cd–Br battery system employing reconstructed sulfate‐based electrolyte.

## Conflicts of Interest

The authors declare no conflicts of interest.

## Supporting information




**Supporting File 1**: supinfo/anie72149‐sup‐0001‐SuppMat.docx.

## Data Availability

The data that support the findings of this study are available from the corresponding author upon reasonable request.
