# Supplementary material for: Highly Reversible Aqueous Anode‐Free Cadmium–Bromine Batteries
Source: Angew Chem Int Ed Engl. 2026 Apr 11;65(21):e1396057. doi: 10.1002/anie.1396057 (PMC13182220; doi:10.1002/anie.1396057)
Supplement: Supplementary file 1 — Supporting File 1: supinfo/anie72149‐sup‐0001‐SuppMat.docx. [file ANIE-65-e1396057-s001.docx]

Supporting Information

**Highly reversible aqueous anode-free cadmium-bromine batteries**

Xun Zhao^+^, Yilong Zhu^+^, Qianru Chen, Rujiao Ma, Junnan Hao*, and Shi-Zhang Qiao*

X. Zhao, Dr. Y. Zhu, Q. Chen, R. Ma, Dr. J. Hao, and Prof. S. Z. Qiao

School of Chemical Engineering, Adelaide University, Adelaide, SA 5005, Australia

^+^ These authors contributed equally to this work.

*Corresponding author: [s.qiao@adelaide.edu.au](mailto:s.qiao@adelaide.edu.au); [junnan.hao@adelaide.edu.au](mailto:junnan.hao@adelaide.edu.au)

**Experimental and Calculational Methods**

**Chemical reagent**: Tetrabutylammonium bromide (Bu_4_NBr, ≥98%), zinc bromide (ZnBr_2_, ≥98%), cadmium sulfate (CdSO_4_, ≥99%), lithium chloride (LiCl, ≥99%), and polytetrafluoroethylene aqueous dispersion (PTFE, 60 *wt.*%) were purchased from Sigma Aldrich. Cadmium bromide (CdBr_2_, 98%) was purchased from ThermoFisher Scientific Australia Pty. Ltd. Zinc sulfate heptahydrate (ZnSO_4_·7H_2_O, 99%) and ethanol (anhydrous, ≥99.5%) were purchased from Chem-Supply Australia Pty. Ltd. Cd foil (500 μm) was purchased from Qinghe County Huiji Metal Materials Co. Ltd. Cu foil (20 μm), Zn foil (50 μm), Ti mesh (60 mesh), and Ketjen Black (KB) were purchased from Canrd New Energy Technology Co. Ltd. Deionized water was used to prepare all aqueous electrolytes.

**Electrolyte preparation**: LiCl (0.424 g, 0.848 g, or 1.272 g) was dissolved in 10 mL of 3 M (mol L^−1^) CdSO_4_ (CSO) aqueous solution to yield electrolyte containing 1 m (mol kg^−1^), 2 m, and 3 m LiCl, denoted as CSO-1LiCl, CSO-2LiCl, and CSO-3LiCl, respectively.

**Preparation of Bu_4_NBr-CdBr_2_ and Bu_4_NBr-ZnBr_2_ electrodes**: Bu_4_NBr-CdBr_2_ and Bu_4_NBr-ZnBr_2_ electrodes were prepared by thoroughly mixing respective active materials with KB conductive additive and PTFE binder. Specifically, the mass ratios used were: Bu_4_NBr:CdBr_2_:KB:PTFE = 3.5:3.5:2:1 and Bu_4_NBr:ZnBr_2_:KB:PTFE = 3.8:3.2:2:1. Ethanol was employed to facilitate uniform mixing. The resulting mixtures were processed into a homogeneous wet dough, which was subsequently pressed onto Ti mesh substrates. The prepared electrodes were dried at 60 °C in air to effectively remove residual water and ethanol. For electrochemical testing in coin cells, the mass loading of the active materials (Bu_4_NBr-CdBr_2_ and Bu_4_NBr-ZnBr_2_) ranged between 5 to 20 mg cm^−2^.

**Electrochemical measurements**: Electrochemical performance of Bu_4_NBr-CdBr_2_ and Bu_4_NBr-ZnBr_2_ cathodes were evaluated using 2032-type coin cells, with glass fiber separator. Cd-Cu batteries were assembled using Cu foil as the cathode, Cd foil as the anode, and CSO-3LiCl electrolyte. Here, anode-free (AF) Cd-Br and Zn-Br batteries were assembled with Bu_4_NBr-CdBr_2_ and Bu_4_NBr-ZnBr_2_ cathodes, respectively, without pre-deposited metal on the anode side (N/P = 0). In these cells, bare Cu foil served as the anode current collector. The specific capacities were calculated based on the mass of Bu_4_NBr-CdBr_2_ or Bu_4_NBr-ZnBr_2_ active materials. For pre-deposited Cu-Zn and Cu-Cd batteries, the anode utilization (AU) is defined as AU = *Q*_strip_ / *Q*_pre-deposited_ × 100%, where *Q*_strip_ is the reversible stripping capacity in each half-cycle and *Q*_pre-deposited_ is the initial amount of metal deposited on the Cu substrate. Accordingly, the 25%, 50%, and 75% AU values discussed for Cu-Zn and Cu-Cd cells correspond to stable cycling in which 25%, 50%, and 75% of the pre-deposited metal is reversibly utilized, respectively. For AF full cells, the design-level AU is 100% because N/P = 0 and no excess Cd or Zn is present at assembly. It is noteworthy that the Cu-Zn batteries and AF Zn-Br batteries exhibit significantly poorer performance in 3 M ZnSO_4_ + 3 m LiCl (ZSO-3LiCl) compared to pure 3 M ZnSO_4_ (ZSO) aqueous solution. Consequently, these AF Zn-based systems utilize ZSO solutions as the electrolyte. Galvanostatic charge-discharge (GCD) tests were carried out using a battery testing system (CT3002A, LAND) within a voltage window of 0.8-1.6 V (*vs*. Cd^2+^/Cd) or 1.0-1.9 V (*vs*. Zn^2+^/Zn). Additionally, linear sweep voltammetry (LSV) and cyclic voltammetry (CV) tests were conducted with a VMP-3 electrochemical workstation. CV measurements were used to investigate Cd stripping/deposition behavior and the redox behaviors in the AF Cd-Br and Zn-Br battery systems. LSV was conducted to obtain the Tafel plots for Zn and Cd electrodes using a scan rate of 1 mV s^−1^, employing a three-electrode configuration with an AgCl/Ag electrode serving as the reference electrode.

**Assembly of AF pouch cells**: The rate capability of AF Cd-Br and Zn-Br batteries was further assessed using pouch cells with a high mass loading of 14 mg cm^−2^. Specifically, AF Cd-Br and Zn-Br pouch cells were assembled utilizing Bu_4_NBr-CdBr_2_ or Bu_4_NBr-ZnBr_2_ cathodes, respectively, each pressed onto a piece of Ti mesh (6 cm × 6 cm). Two pieces of Cu foil, slightly larger than the cathode dimensions, served as the current collectors. CSO-3LiCl or ZSO were used as the electrolyte. All pouch cells were hermetically sealed using a vacuum sealing machine (MSK-115A-S).

**Differential electrochemical mass spectrometry (DEMS)**: Hydrogen generation during Zn and Cd deposition was *in situ* monitored using an online DEMS system (HPR-40, HIDEN) integrated with a customized electrochemical cell. The hydrogen produced during the electrodeposition process was pumped into the vacuum chamber for the subsequent mass spectrometric analysis. Following previously reported methods, an Au film (thickness: 50 μm) sputtered onto a PTFE membrane with a pore size of 0.02 μm served as the working electrode, while a graphite rod and AgCl/Ag electrode were employed as the counter electrode and reference electrode, respectively. LSV measurements were performed using an electrochemical workstation (CHI760E, Chenhua) at a scan rate of 5 mV s^−1^.

***In situ* electrochemical gas chromatography (EC-GC) measurements**: The hydrogen evolution reaction (HER) of the symmetric Zn and Cd batteries during the charging and discharging process was monitored via *in situ* EC-GC. GC (Agilent, 8890B) with a thermal conductivity detector was used to collect and detect the produced gas, where argon with a flow rate of 10 mL min^−1^ was used as the carrier gas. As shown in **Figure S2**, a two-electrode electrolytic cell was used, where Zn or Cd foils were used as the electrodes with a size of 3 cm × 1.8 cm and an effective area of 4.5 cm^2^ and 20 mL of ZSO or CSO aqueous solution was used as electrolyte. GCD tests of the symmetric batteries were conducted on a LAND battery testing system, where the current density was set at 2 mA cm^−2^.

**Characterization techniques**: The surface roughness of cycled Cd foils was analyzed using 3D confocal microscopy (Olympus LEXT OLS5000 Profilometer). Raman spectra were acquired using an inVia confocal Raman microscope (Renishaw) with a 532 nm laser source. Fourier transform infrared spectra were collected using a Thermo Scientific Nicolet iS50 spectrometer. X-ray diffraction patterns of cycled Cd and Zn foils were obtained using a Rigaku MiniFlex 600 diffractometer. Surface morphologies of deposited Zn and Cd on Cu foils were examined by an ultra-high-resolution scanning electron microscope (SEM, Hitachi SU7000).

**Safety and environmental note:** Cd-containing chemicals and electrodes used in this work are toxic and must be handled with appropriate precautions. All preparation, cell assembly, disassembly, and post-test handling of Cd-containing materials were conducted in accordance with institutional hazardous-material safety protocols, including the use of suitable personal protective equipment and designated waste collection procedures. All electrochemical tests were conducted in sealed cell configurations without leakage or environmental exposure. All test cells, as well as Cd-containing solid and liquid wastes, were collected and disposed of as hazardous waste following the relevant institutional and regulatory requirements. Therefore, the present study should be regarded as a research proof-of-concept for demonstrating the feasibility of an aqueous AF Cd-Br battery chemistry.

**Calculation methods**: The classic molecular dynamics (MD) simulations of CSO and CSO-3LiCl systems were conducted to investigate the solvation shell structure of Cd^2+^ using the Nanoscale Molecular Dynamics package.^[1]^ The solution model contained different numbers of water molecules and salts, as shown in **Table S1**. The force field parameters for each group (water, ions, and anions) were obtained from CHARMM36 force fields.^[2]^ The TIP3P water model was employed for water.^[3]^ The time step was set to be 2 fs. The cutoff radius for vdW and electrostatic interactions were 12 and 10 Å, respectively. The standard periodic boundary condition was used in all simulations. After minimization of the initial structure for 5,000 steps (10 ps), each system was heated from 100 to 300 K by performing Langevin dynamics temperature control for 0.8 ns. The systems were further relaxed for another 9.2 ns under the NPT ensemble using the Nosé-Hoover Langevin piston pressure control method at 1.013125 bar.^[4]^ After relaxation, each system was simulated for 50 ns under the canonical ensemble (NVT) for data collection and statistical analysis.^[5]^ The periodic boundary cell sizes are shown in **Table S2**. The visualization of Cd^2+^ solvation shell structure in CSO and CSO-3LiCl, as well as the analyses of radial distribution function (RDF) and electrostatic potential (ESP) of CdCl^+^ and Cd^2+^, were performed using Visual Molecular Dynamics (VMD, version 1.9.3). Adsorption energy (*E*_ads_) was computed based on the energy difference of these optimized structures according to the following equation:

*E*_ads_ = *E*_tot_ – *E*_1_ – *E*_2_

where *E*_tot_ represents the total energy of Cu-CdCl^+^ or Cu-Cd^2+^ complex, and *E*_1_ and *E*_2_ denote the energies of CdCl^+^/Cd^2+^ and Cu substrate, respectively. Similarly, charge density difference of Cu-CdCl^+^ or Cu-Cd^2+^ complex was obtained using Visualization for Electronic and Structural Analysis (VESTA, version 3.90.3a) according to the following equation:

Δ*ρ* = *ρ*_tot_ – *ρ*_1_ – *ρ*_2_

where *ρ*_tot_ represents the total electron density of Cu-CdCl^+^ or Cu-Cd^2+^ complex, and ρ_1_ and ρ_2_ represent the electron densities of CdCl^+^/Cd^2+^ and Cu substrate, respectively. The isosurface level for visualization was set to 0.001.


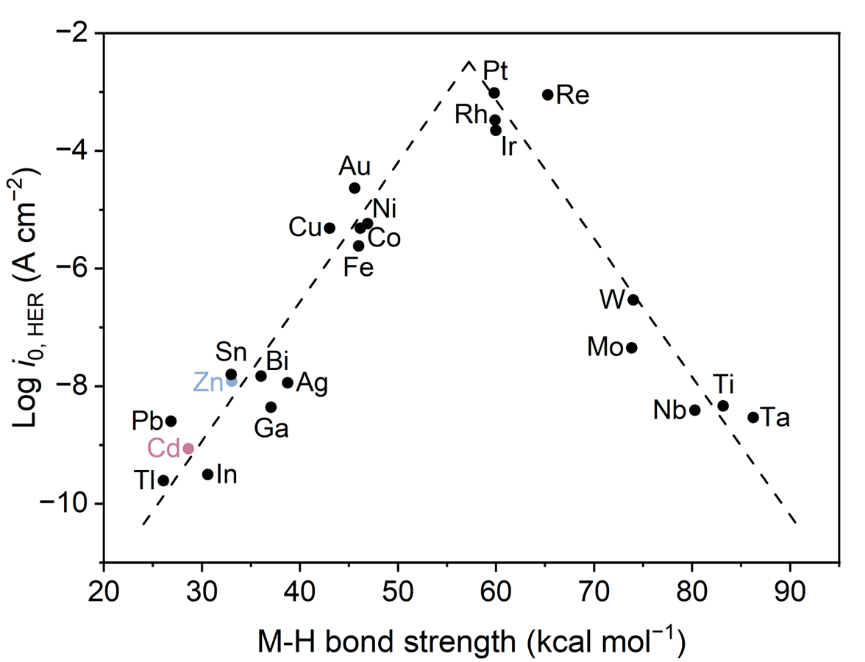


**Figure S1.** A volcano plot of different metals for HER, illustrating the relationship between the exchange current densities for electrolytic HER and the strength of the metal-hydrogen bond formed during the electrochemical reaction, reproduced from Ref. [6], Copyright (1972), with permission from Elsevier. The volcano plot follows the Sabatier principle, showing that metals with moderate M-H bond strength (such as Pt, Rh, and Ir) exhibit the highest HER exchange current densities because they provide optimal hydrogen adsorption and desorption energetics. Metals on the left with weak M-H bonding (*e.g*. Zn, Cd, and Pb) adsorb hydrogen too weakly, limiting H* formation, while those on the right with overly strong M-H bonding (*e.g*. Ti, Nb, Ta) bind hydrogen too tightly, hindering H_2_ desorption, thus both leading to slower HER kinetics.


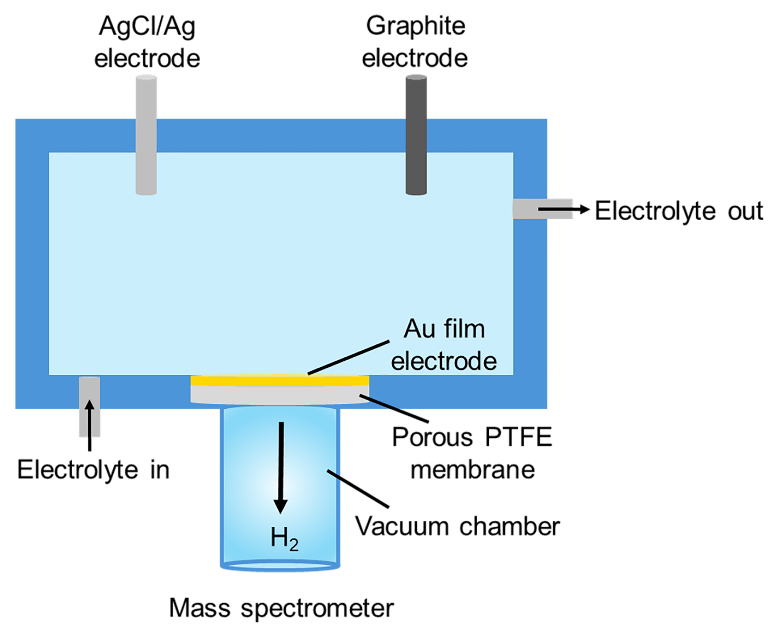


**Figure S2**. Schematic of the electrochemical cell for DEMS measurement. The Au-coated PTFE membrane serves as the working electrode, allowing H_2_ generated during deposition to pass into the vacuum chamber for mass spectrometric detection while blocking liquid electrolyte.


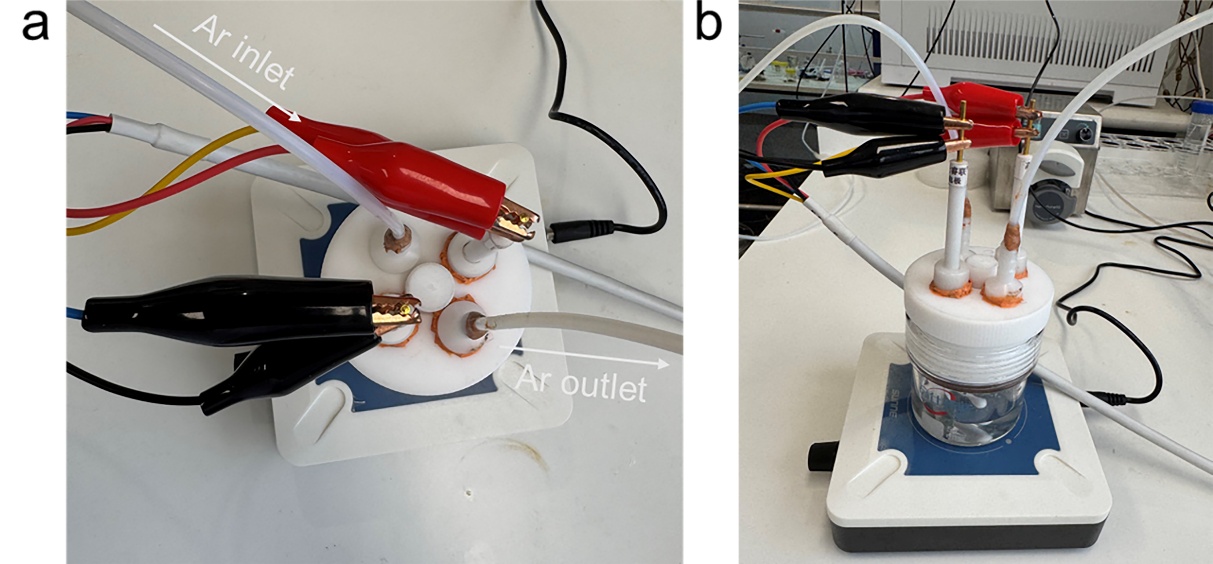


**Figure S3.** Photographs of the two-electrode electrolytic cell used for *in situ* EC-GC measurements, shown in (a) top and (b) side views. The sealed cell is charged with ZSO or CSO electrolyte and contains a magnetic stir bar; measurements are performed with the cell placed on a magnetic stirrer. The lid integrates two electrode clamps and two gas parts to provide continuous purge flow and maintain an inert atmosphere during Zn^2+^/Cd^2+^ deposition.


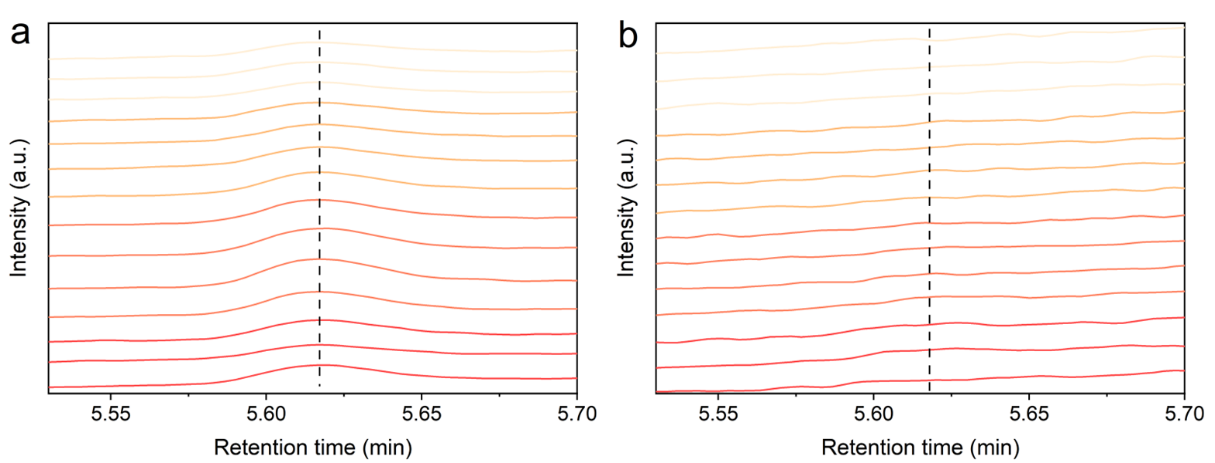


**Figure S4.** Stacked *in situ* EC-GC profiles of symmetric (a) Zn and (b) Cd batteries at 2 mA cm^−2^. The symmetric Zn cell exhibits a distinct hydrogen signal during cycling, whereas the Cd cell shows nearly no detectable gas signal.


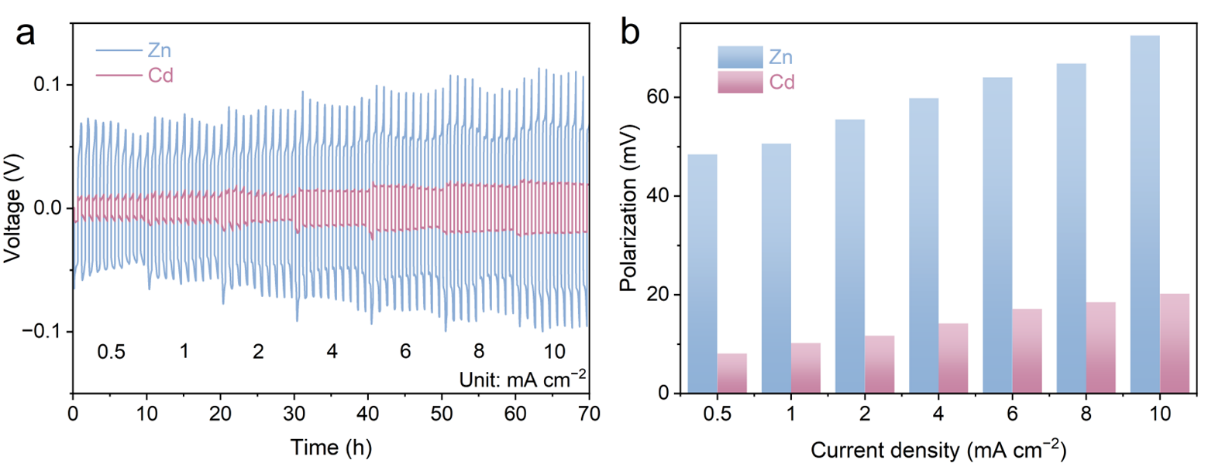


**Figure S5.** (a) Rate performance of symmetric Zn and Cd batteries. (b) The voltage hysteresis of Zn and Cd anodes. At all current densities, Cd metal exhibits significantly lower polarization than Zn.


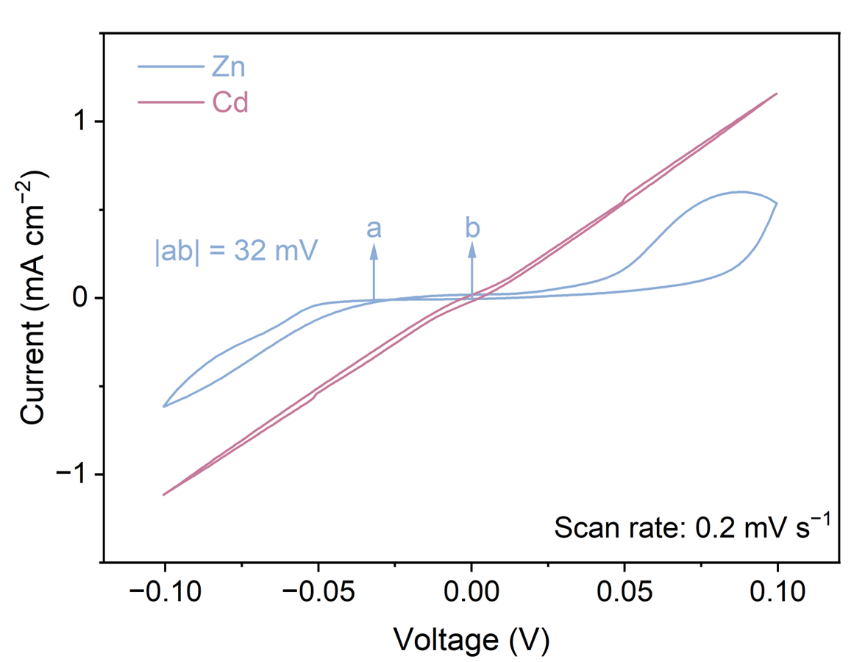


**Figure S6.** CV curves of symmetric Zn and Cd batteries at a scan rate of 0.2 mV s^−1^. The symmetric Zn cell shows a clear polarization of 32 mV, while the Cd cell responds almost instantly to voltage changes with negligible polarization.


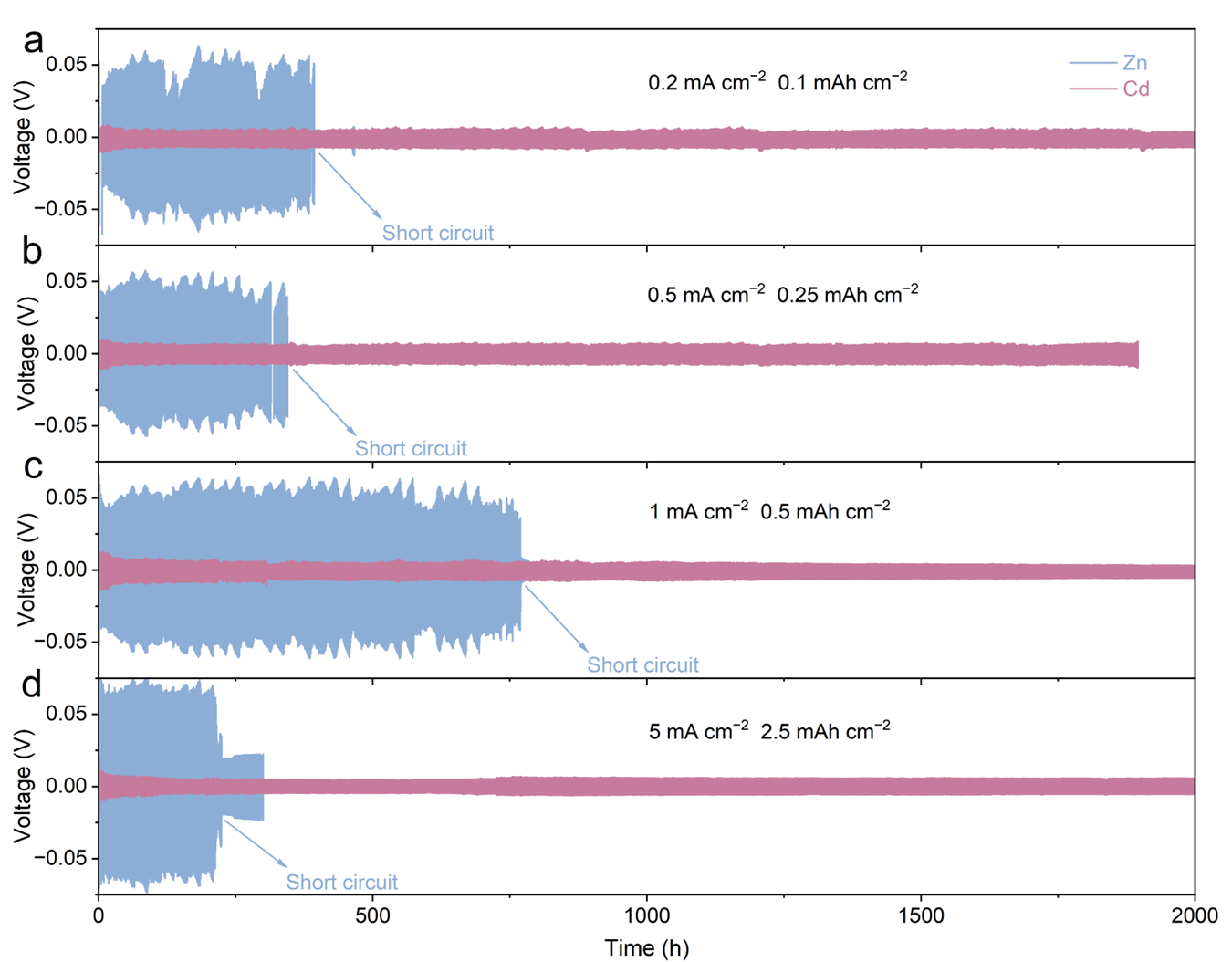


**Figure S7.** Cyclic stability of symmetric Zn and Cd batteries at (a) 0.2 mA cm^−2^/0.1 mAh cm^−2^, (b) 0.5 mA cm^−2^/0.25 mAh cm^−2^, (c) 1 mA cm^−2^/0.5 mAh cm^−2^, and (d) 5 mA cm^−2^/2.5 mAh cm^−2^. The symmetric Zn cells short-circuit rapidly even at low current densities, whereas the Cd cells remain stable for over 2,000 h.


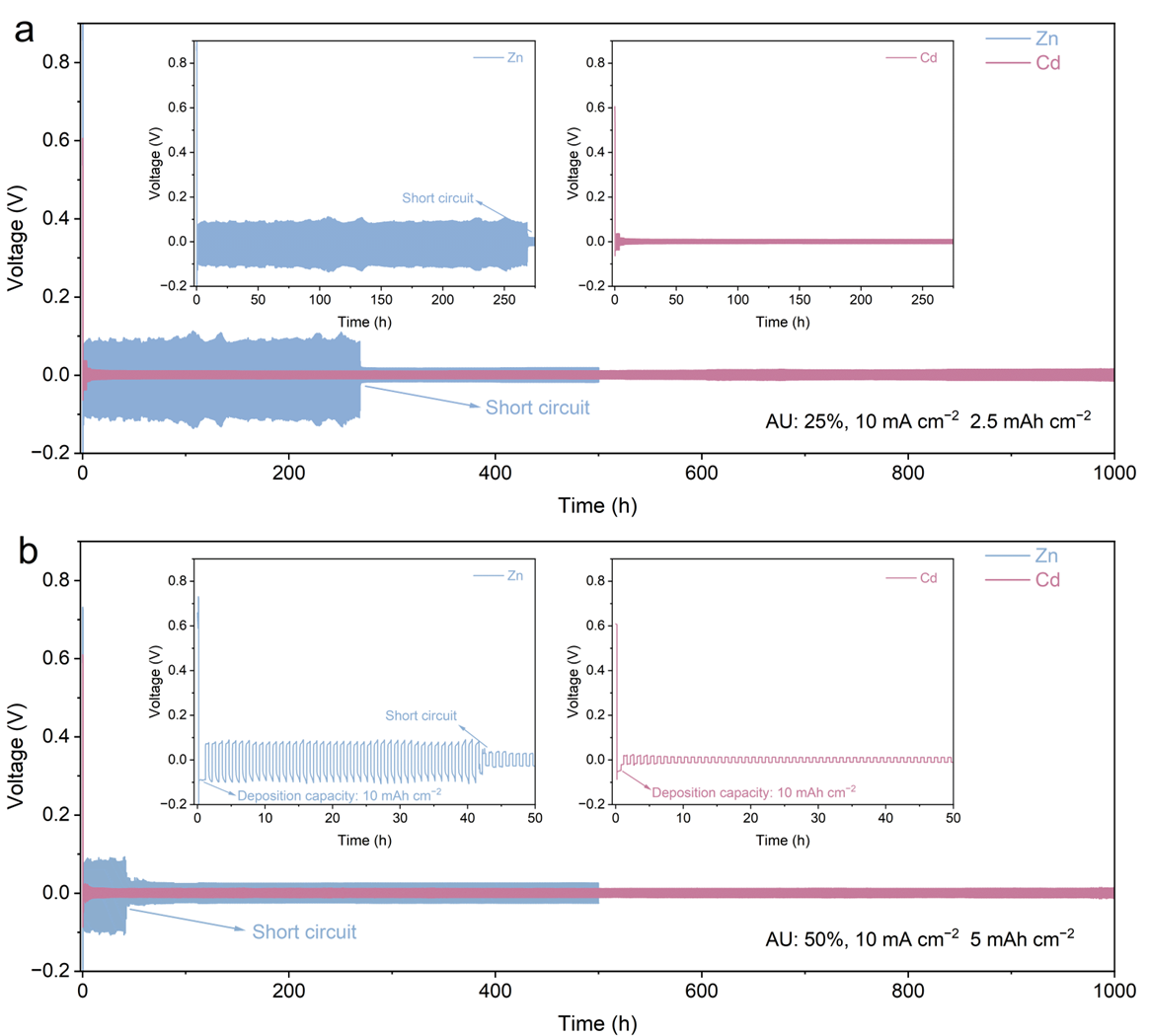


**Figure S8.** Cyclic stability of Cd-Cd and Zn-Zn batteries at a current density of 10 mA cm^−2^ and AU of (a) 25% and (b) 50%. Under high AU and current density, the Zn cells short-circuit rapidly, whereas the Cd cells remain stable for over 1,000 h of continuous cycling.


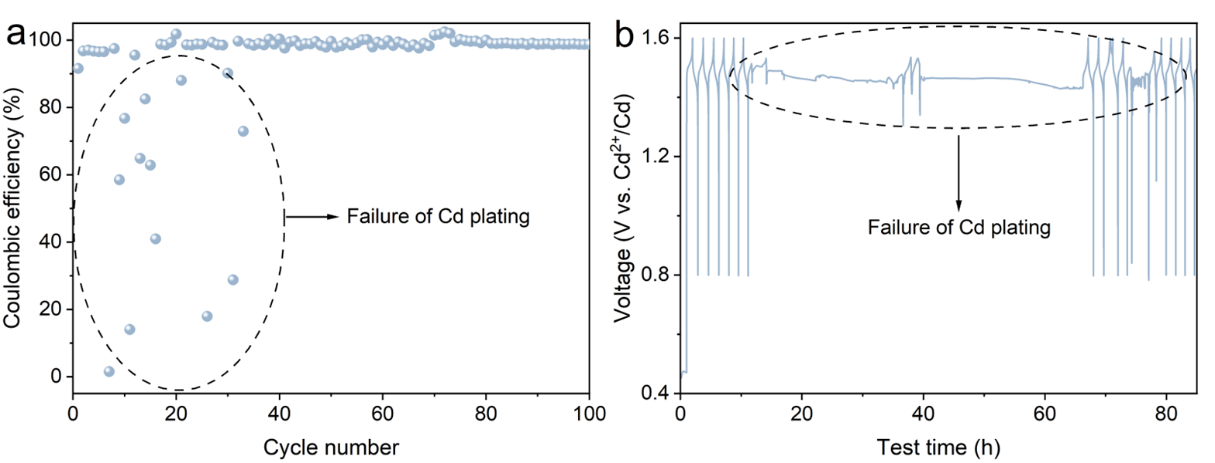


**Figure S9.** (a) Coulombic efficiency and (b) GCD profiles of AF Cd-Br batteries using CSO as electrolyte at 1 C. The AF Cd-Br batteries using pure CSO electrolyte show fluctuating Coulombic efficiency due to the sluggish Cd^2+^ deposition kinetics.


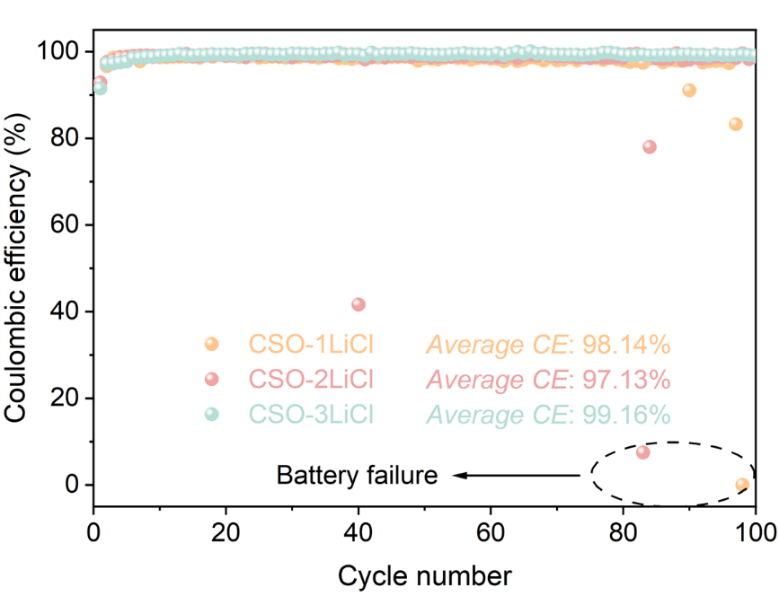


**Figure S10.** Comparison of Coulombic efficiency of AF Cd-Br batteries using different CSO-*x*LiCl electrolytes at 1 C. CSO-1LiCl and CSO-2LiCl electrolytes exhibit noticeable Coulombic efficiency fluctuations followed by battery failure, whereas the CSO-3LiCl electrolyte enables stable cycling of AF Cd-Br batteries for 100 cycles.


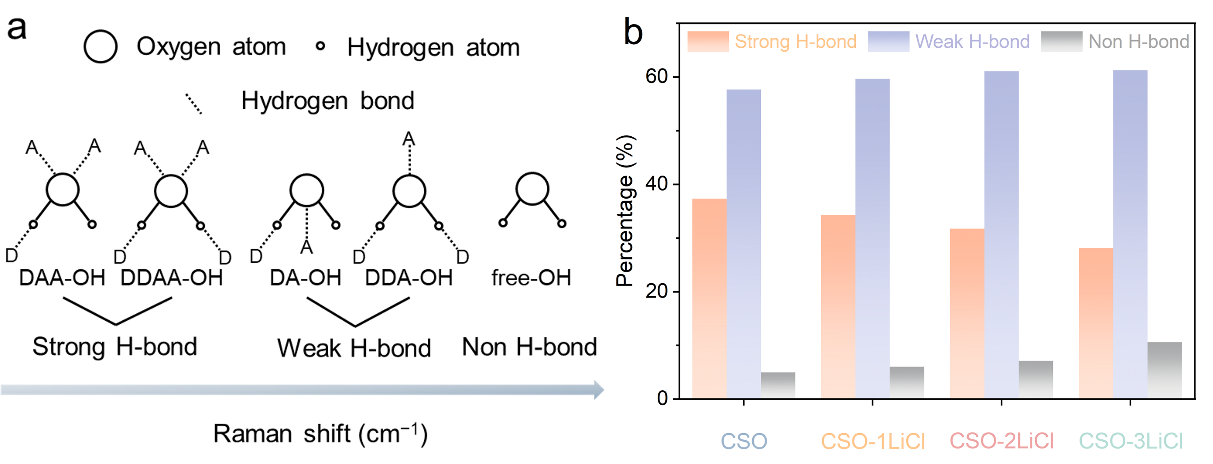


**Figure S11.** (a) Schematic illustration of different types of hydrogen bonds based on the proton donor-acceptor (D-A) model. (b) The calculated percentage of strong, weak, and non H-bonds of CSO and CSO-*x*LiCl. With increasing LiCl concentration, the fractions of weak and non H-bonds increase, whereas that of strong H-bond decreases, indicating progressive disruption of the water H-bond network and thereby improving Cd^2+^ deposition kinetics.


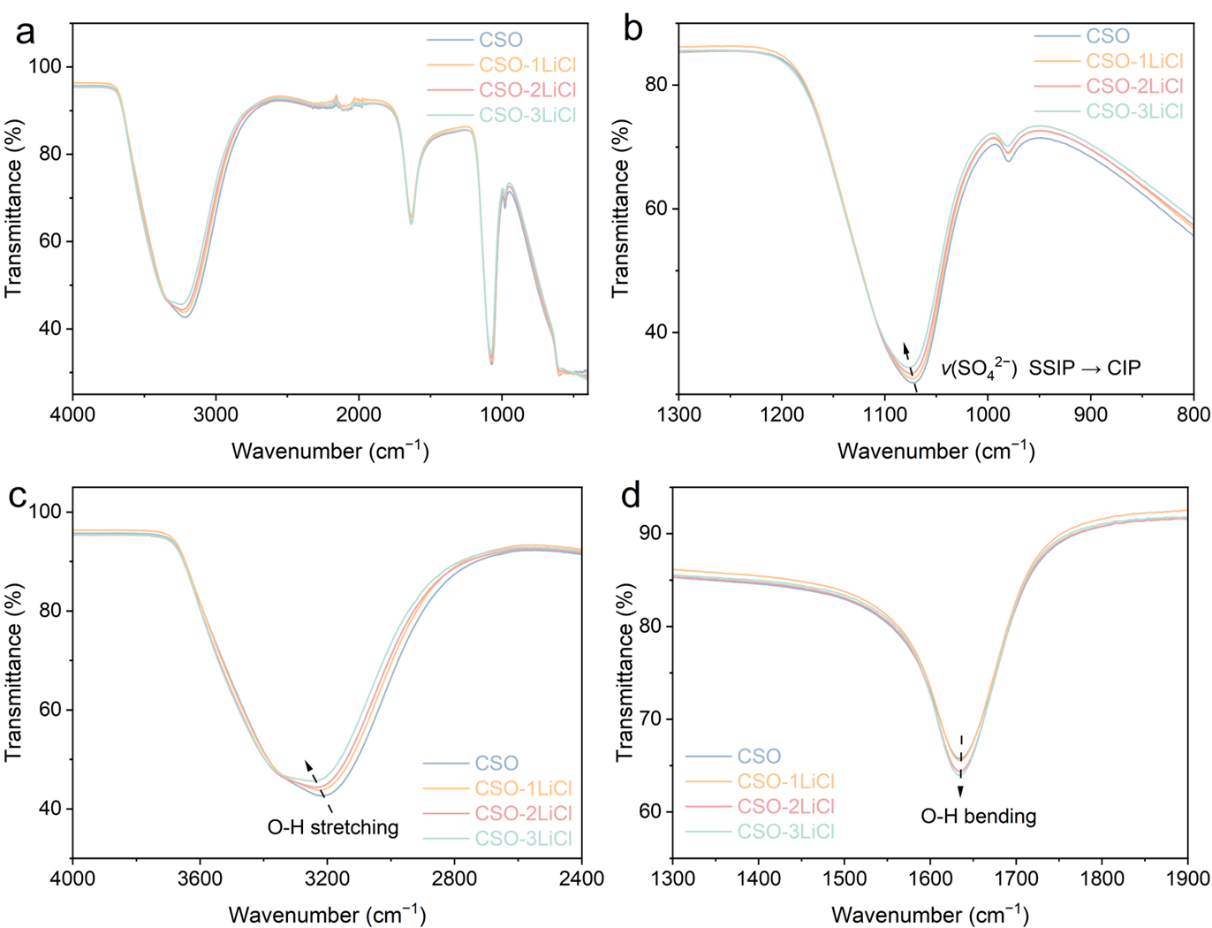


**Figure S12.** (a) FTIR spectra of CSO and CSO-*x*LiCl, with enlarged views of (b) the symmetric SO_4_^2−^ stretching region, (c) the O-H stretching region, and (d) the O-H bending region. The vibrations in these characteristic bands further confirm that the addition of LiCl alters the H-bond network within the electrolyte.


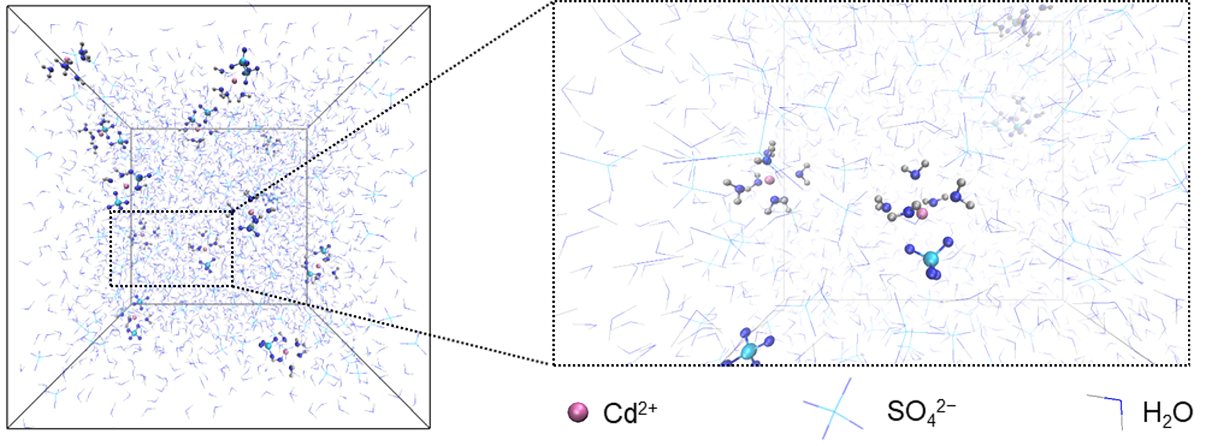


**Figure S13.** 3D snapshot of CSO system from MD simulations and partial enlarged snapshot. The enlarged snapshot clearly reveals the presence of typical Cd[(H_2_O)_6_] and Cd[SO_4_(H_2_O)_5_] solvation structures within the CSO system.


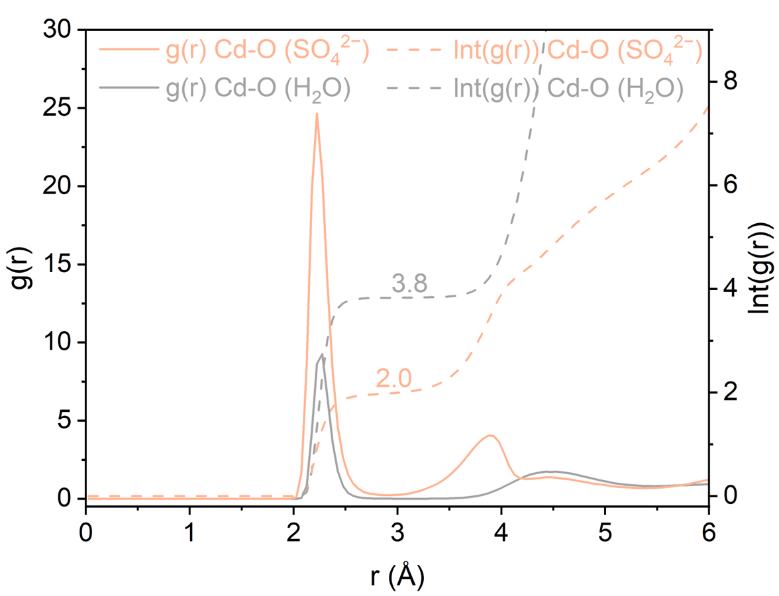


**Figure S14.** RDF (g(r) and lnt(g(r))) for Cd-O (SO_4_^2−^) and Cd-O (H_2_O) from MD simulations of CSO. The coordination numbers of Cd-O (SO_4_^2−^) and Cd-O (H_2_O) are 2.0 and 3.8, respectively.


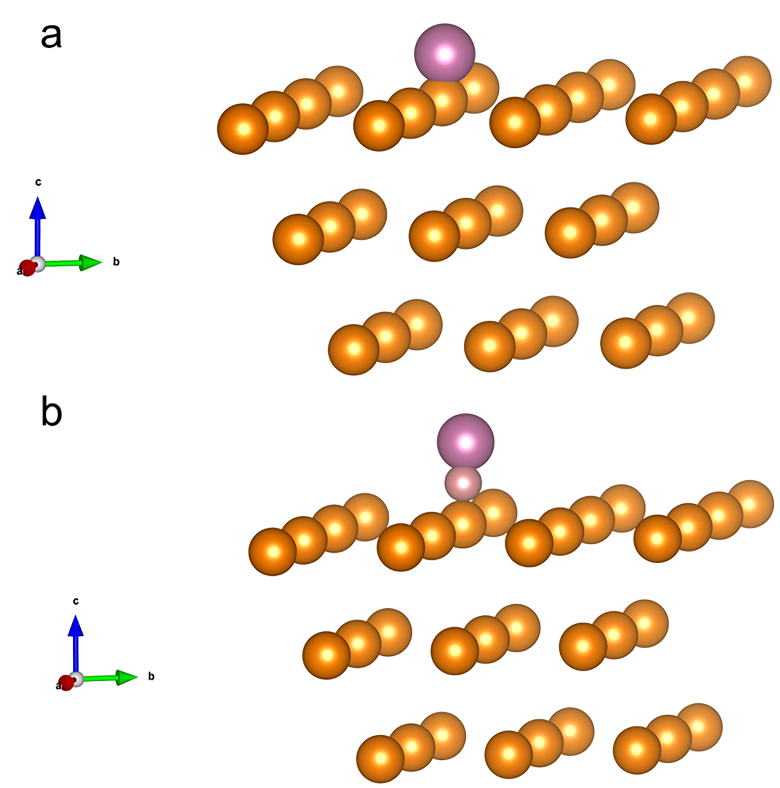


**Figure S15.** Optimized adsorption configurations of (a) Cu-Cd^2+^ and (b) Cu-CdCl^+^ used for adsorption energy calculations and charge density difference analysis. The Cu-CdCl^+^ configuration binds strongly to Cu substrate, exhibiting the most favorable adsorption energy. Participation of Cl^−^ in the Cd^2+^ solvation shell leads to the formation of CdCl^+^, which may be reduced as a diatomic species on Cu, thereby accelerating Cd^2+^ deposition kinetics.


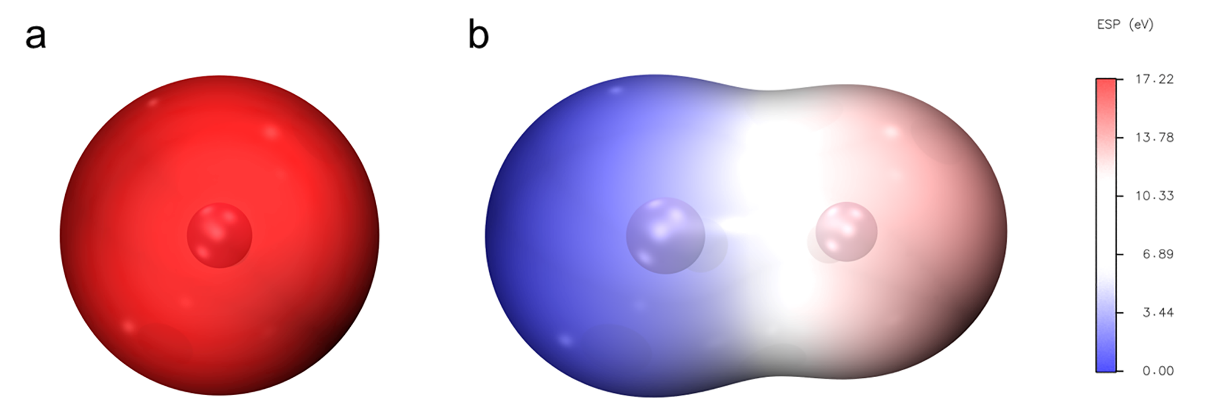


**Figure S16.** ESP of (a) Cd^2+^ and (b) CdCl^+^. CdCl^+^ shows a substantially attenuated positive potential at the molecular surface, consistent with weaker Lewis acidity due to Cl^−^ coordination.


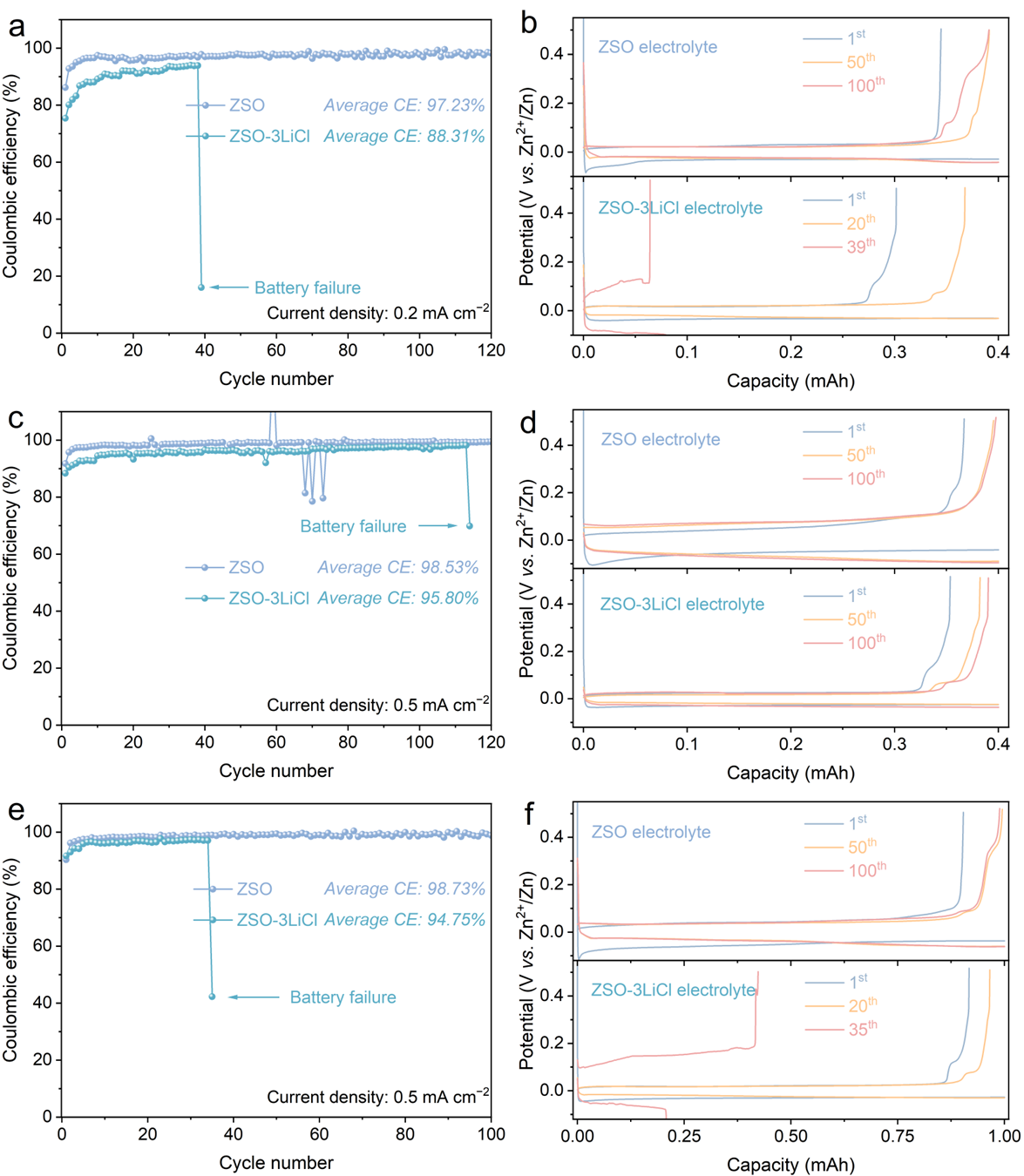


**Figure S17**. Coulombic efficiency and GCD curves of Cu-Zn batteries using ZSO or ZSO-3LiCl electrolyte at (a, b) 0.2 mA cm^−2^/0.2 mAh cm^−2^, (c, d) 0.5 mA cm^−2^/0.2 mAh cm^−2^, and (e, f) 0.5 mA cm^−2^/0.5 mAh cm^−2^.


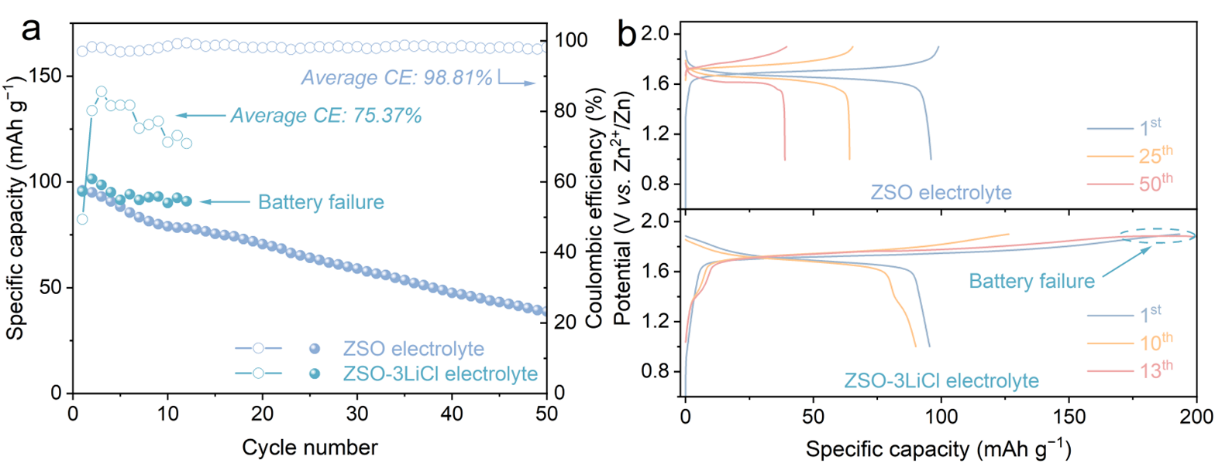


**Figure S18**. (a) Coulombic efficiency and (b) GCD curves of AF Zn-Br batteries using ZSO or ZSO-3LiCl electrolyte at 1 C. Coulombic efficiency comparisons and GCD curves of Cu-Zn (**Figure S17**) and AF Zn-Br batteries (**Figure S18**) using ZSO or ZSO-3LiCl as electrolyte reveal that ZSO consistently delivers higher Coulombic efficiency and better cycling stability, whereas ZSO-3LiCl results in rapid cell failure, underscoring ZSO as the more favorable electrolyte for AF Zn-based batteries.


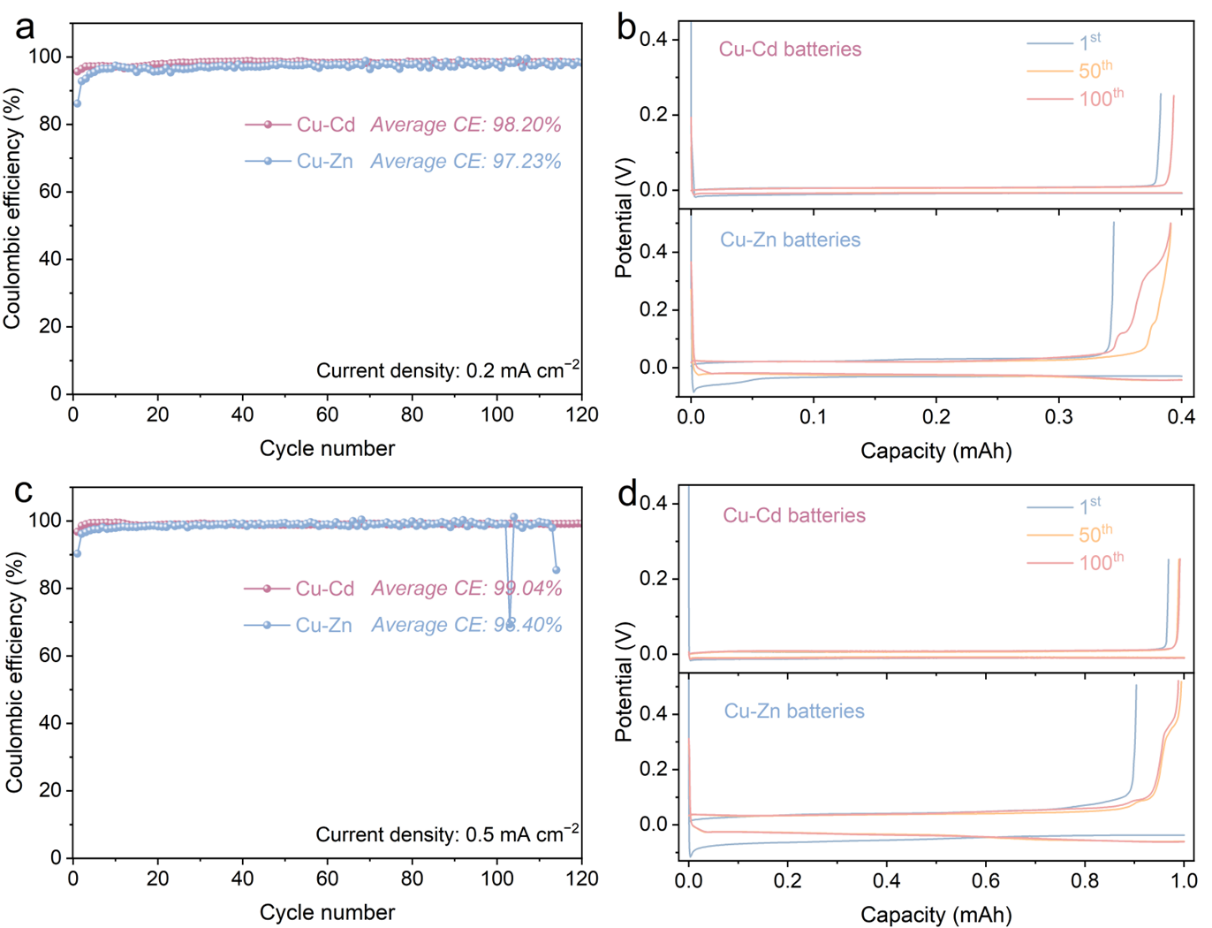


**Figure S19.** Coulombic efficiency and GCD curves of Cu-Cd and Cu-Zn batteries at (a, b) 0.2 mA cm^−2^/0.2 mAh cm^−2^ and (c, d) 0.5 mA cm^−2^/0.5 mAh cm^−2^. Under both conditions, Cu-Cd batteries exhibit higher average Coulombic efficiency and markedly lower polarization than Cu-Zn batteries, indicating more reversible plating/stripping.


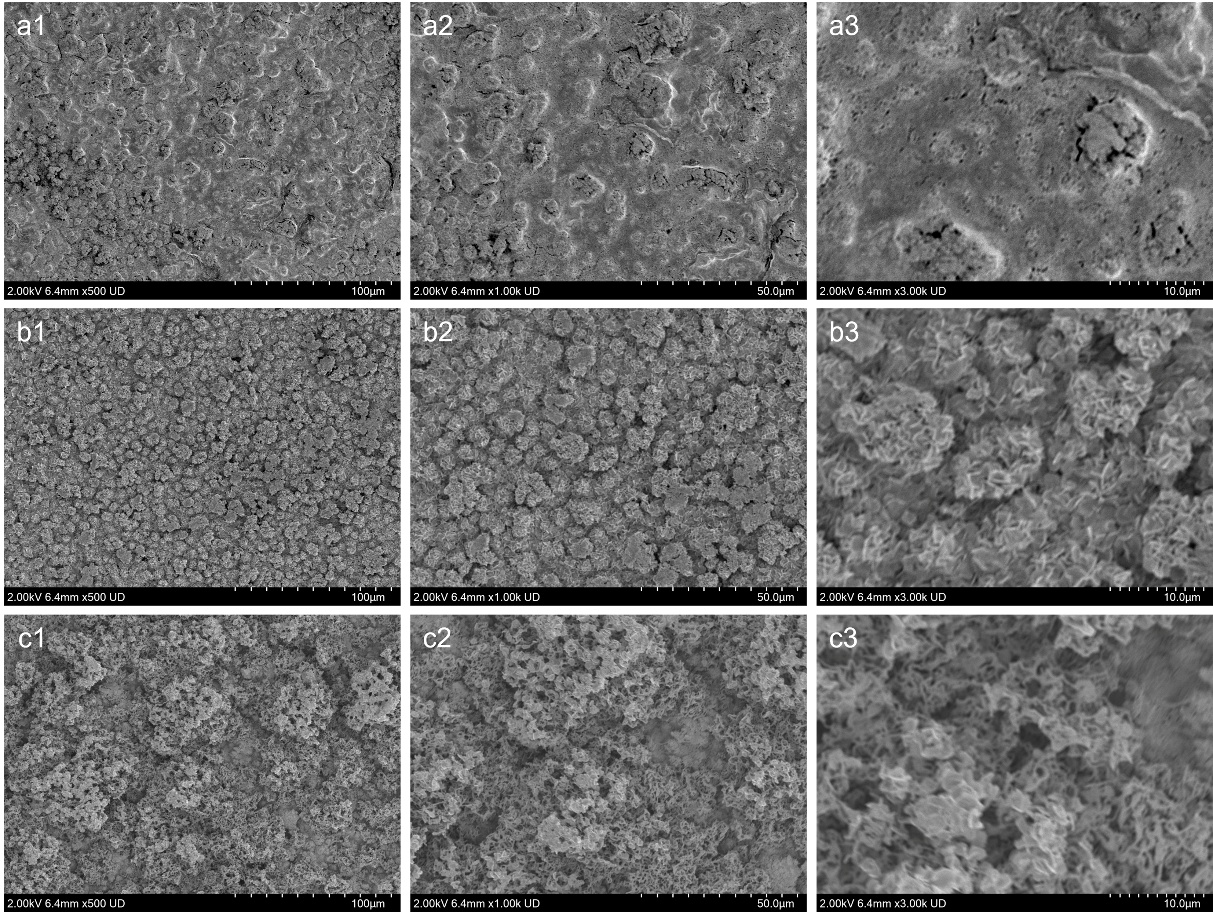


**Figure S20.** SEM images of Zn@Cu at a deposited capacity of (a) 0.5, (b) 1, and (c) 2 mAh cm^−2^. The deposited Zn exhibits typical mossy structures accompanied by byproducts.


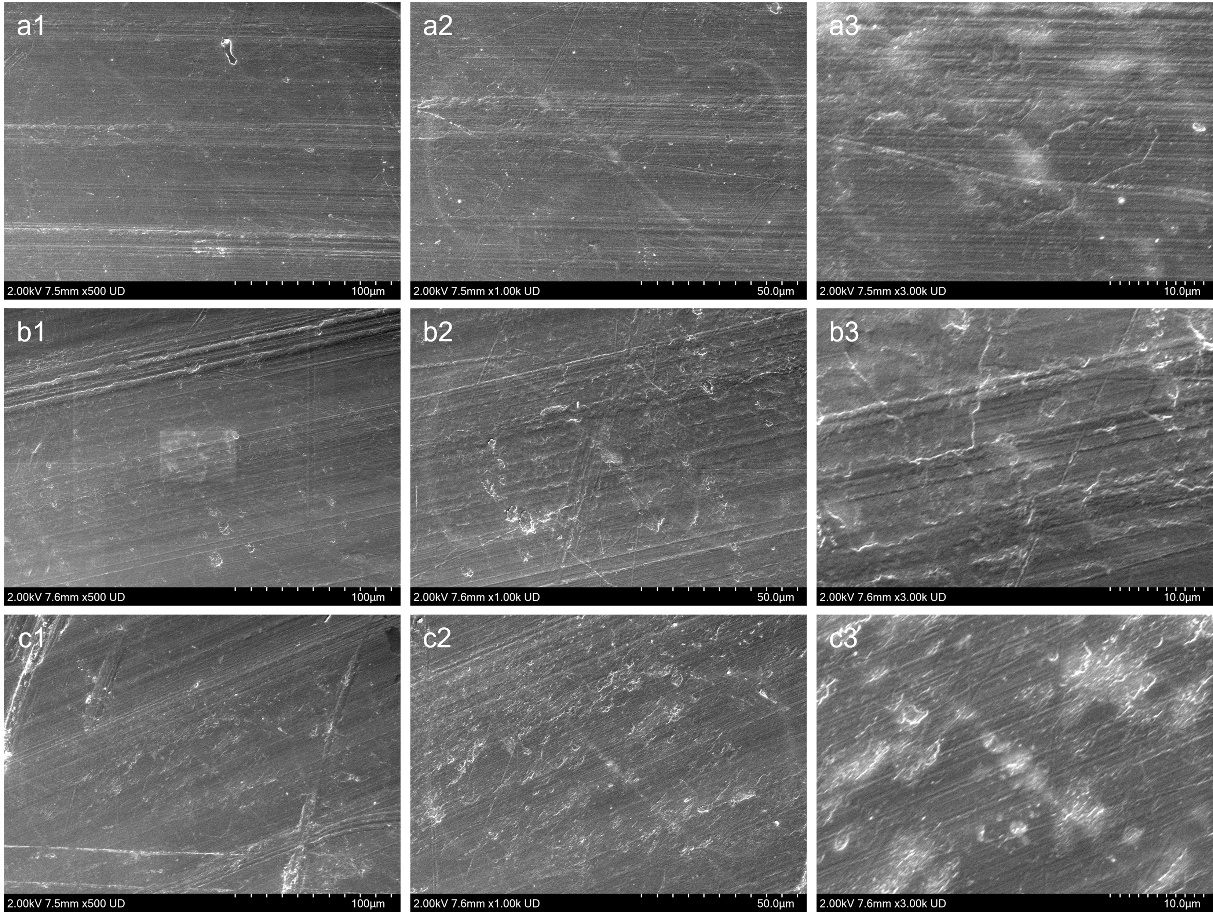


**Figure S21.** SEM images of Cd@Cu at a deposition capacity of (a) 0.5, (b) 1, and (c) 2 mAh cm^−2^. The deposited Cd exhibited uniform, dense, and compact morphologies.


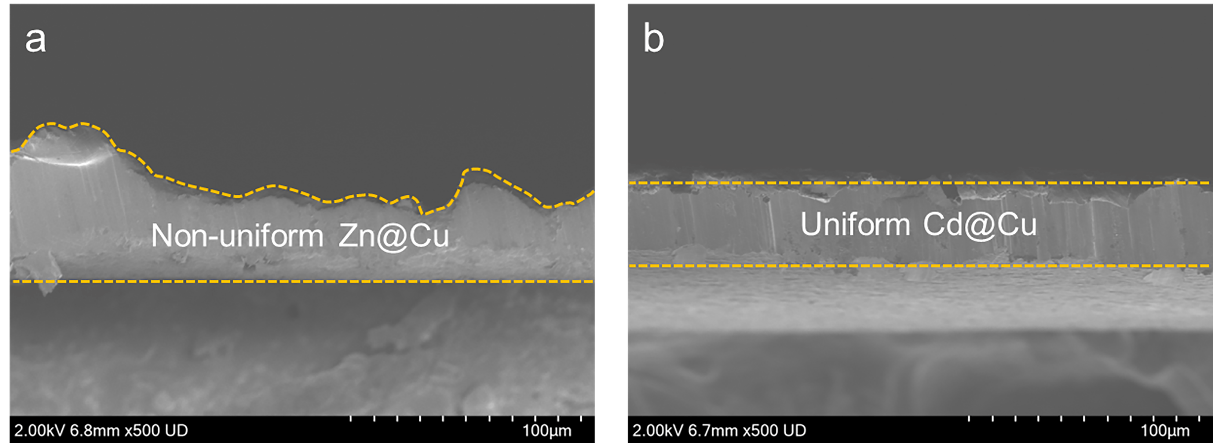


**Figure S22.** Cross-section SEM images of (a) Zn@Cu and (b) Cd@Cu after depositing 5 mAh cm^−2^. The deposited Zn layer is uneven and rough, whereas the Cd layer is markedly flatter and more uniform.

**
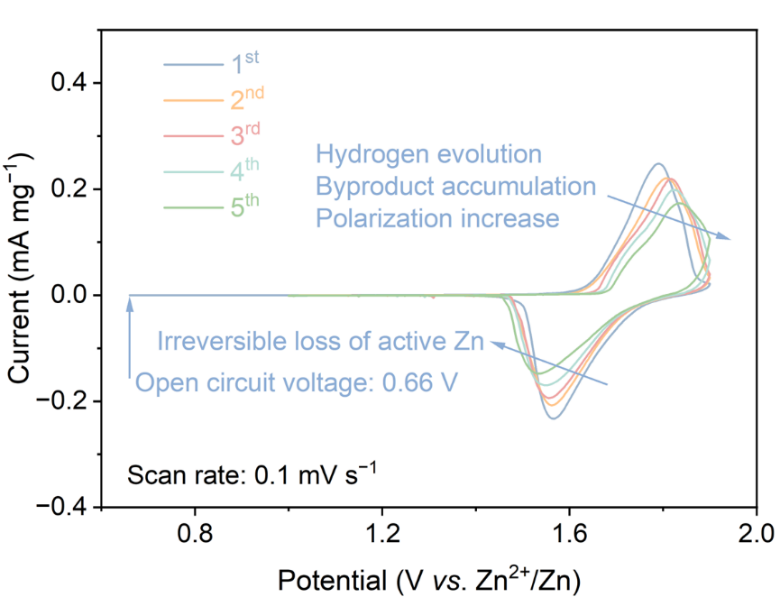
**

**Figure S23.** CV curves of AF Zn-Br coin cells at a scan rate of 0.1 mV s^−1^. The cells exhibit an open-circuit voltage of 0.66 V. During Zn^2+^ deposition, HER and the accumulation of by-products rapidly increase polarization and cause an irreversible loss of active Zn.

**
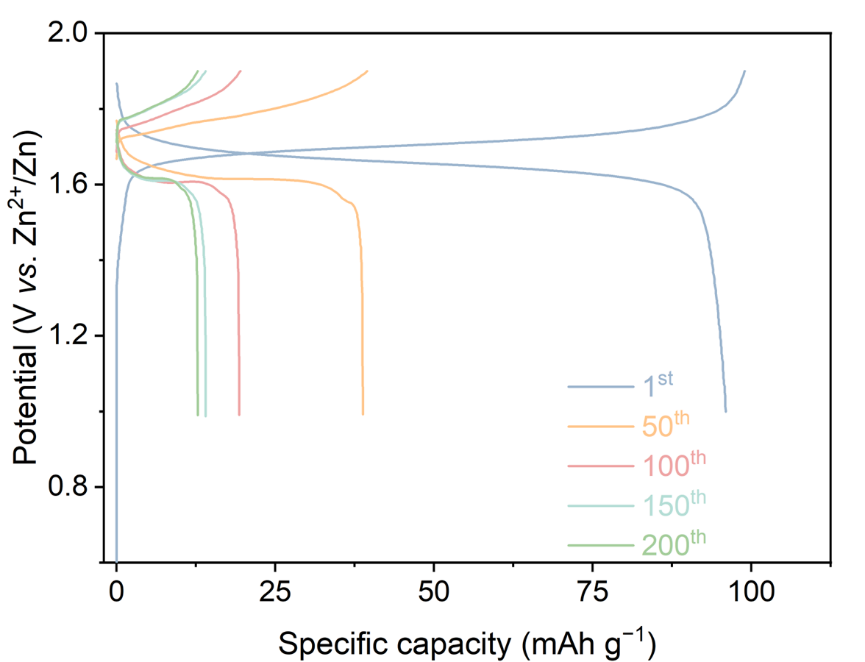
**

**Figure S24.** GCD curves of AF Zn-Br coin cells at 1 C after various cycle numbers. The cells exhibit pronounced capacity fade at 1 C, driven by the rapid depletion of active Zn during cycling.


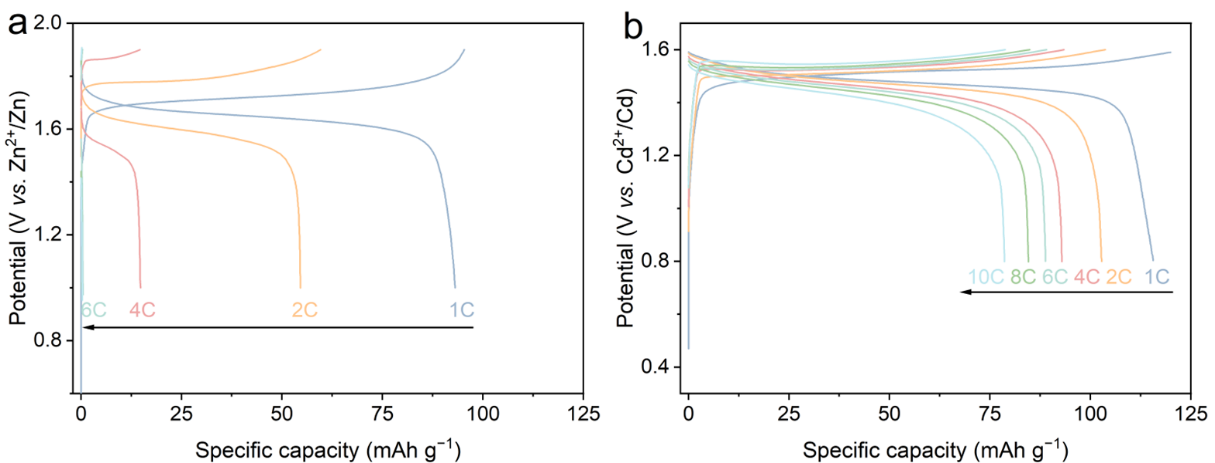


**Figure S25.** GCD curves of AF (a) Zn-Br and (b) Cd-Br coin cells measured at rates from 1 C to 10 C. The AF Zn-Br cell suffers rapid capacity fade already at 1-2 C, leaving negligible capacity at higher rates; by contrast, the AF Cd-Br cell retains ~80 mAh g^−1^ even at 10 C, indicating markedly superior rate capability.


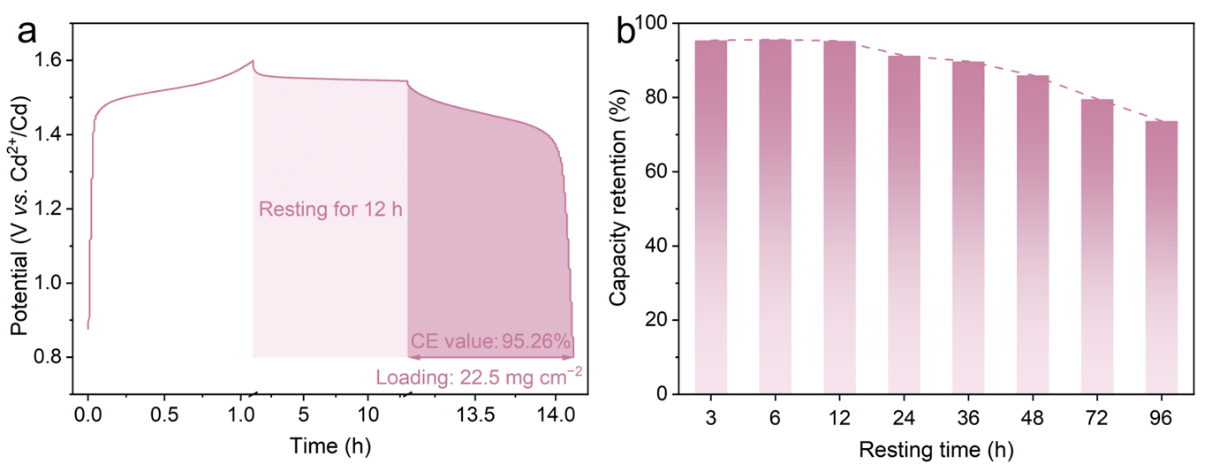


**Figure S26.** (a) GCD profiles of AF Cd-Br batteries at 1 C after a 12 h rest and (b) capacity retention versus resting time. The AF Cd-Br cell with a high mass loading of 22.5 mg cm^−2^, charged at 1 C, retains 95.26% of its capacity after 12 h of rest and nearly 80% after 72 h, indicating low self-discharge.

**
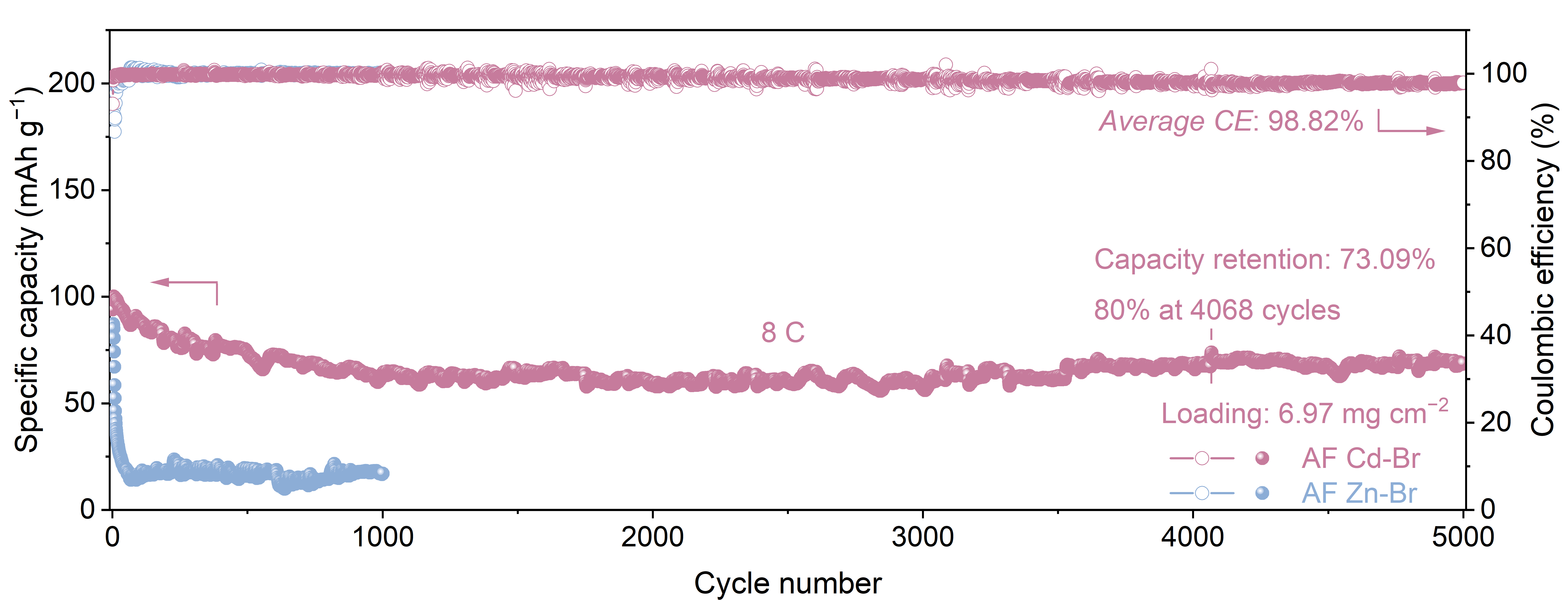
**

**Figure S27.** Long-term cycling of AF Cd-Br and Zn-Br coin cells at a rate of 8 C. The AF Cd-Br cell with a high mass loading of 6.97 mg cm^−2^ retains 80% of its capacity after 4,000 cycles and 73.09% after 5,000 cycles, with an average Coulombic efficiency of 98.82%. In contrast, the AF Zn-Br cell undergoes rapid capacity decay, dropping sharply within the first 50 cycles.


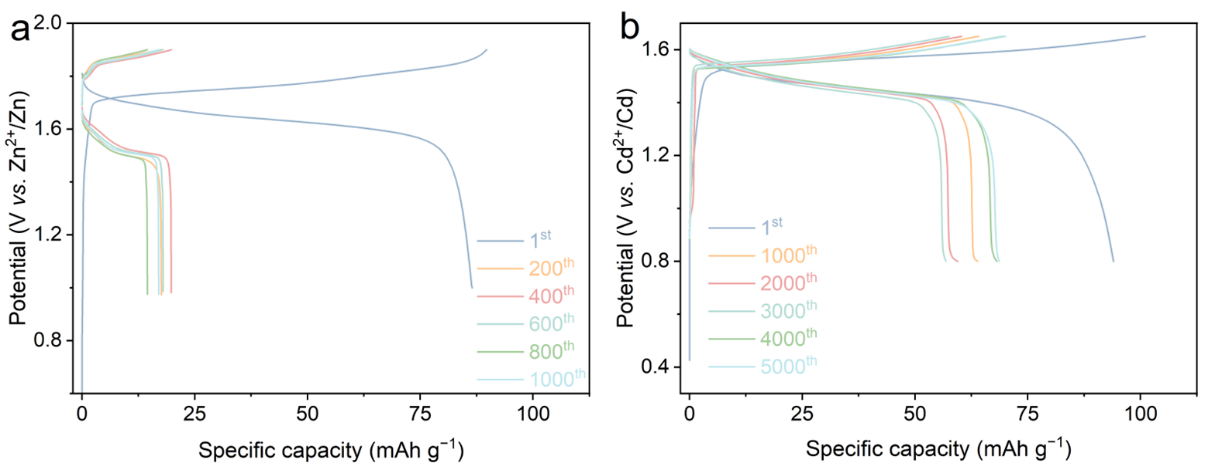


**Figure S28.** GCD curves of AF (a) Zn-Br and (b) Cd-Br coin cells at 8 C. While the AF Zn-Br cell undergoes rapid capacity fade within the first 50 cycles, the AF Cd-Br cell maintains high capacity retention even after 5,000 cycles, underscoring its superior high-rate durability.


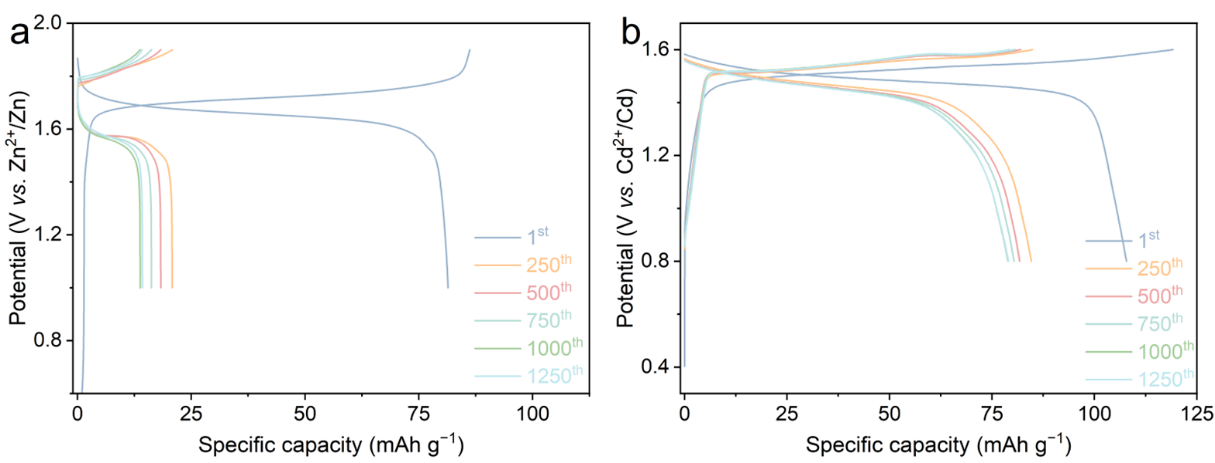


**Figure S29.** GCD curves of AF (a) Zn-Br and (b) Cd-Br pouch cells at 1 C. The AF Zn-Br pouch cell undergoes rapid capacity fade within the first 100 cycles, whereas the AF Cd-Br pouch cell retains 83.8% of its initial capacity after 1,250 cycles, demonstrating markedly superior cycling stability.

**Table S1.** Numbers of molecules in CSO and CSO-3LiCl solution models

| System | Number of H_2_O | Number of CdSO_4_ | Number of LiCl |
| --- | --- | --- | --- |
| CSO | 2540 | 200 | 0 |
| CSO-3LiCl | 2540 | 200 | 200 |

**Table S2.** Periodic boundary size of the CSO and CSO-3LiCl systems

| System | Cell length (Å) | Cell length (Å) | Cell length (Å) |
| --- | --- | --- | --- |
| CSO | 43.16 | 43.16 | 43.16 |
| CSO-3LiCl | 44.41 | 44.41 | 44.41 |

**Table S3.** Statistics analysis of Cd^2+^ solvation structures of CSO with a coordination distance ≤ 3 Å

| Solvation structures | Number | Percentage |
| --- | --- | --- |
| Cd[(SO_4_)_2_(H_2_O)_4_] | 62 | 42.5% |
| Cd[(SO_4_)_2_(H_2_O)_3_] | 28 | 19.2% |
| Cd[SO_4_(H_2_O)_5_] | 25 | 17.1% |
| Cd[(SO_4_)_3_(H_2_O)_2_] | 17 | 11.6% |
| Cd[(SO_4_)_3_(H_2_O)_3_] | 5 | 3.4% |
| Cd[(SO_4_)_3_H_2_O] | 5 | 3.4% |
| Cd[(H_2_O]_6_] | 2 | 1.4% |
| Cd[(SO_4_)_4_H_2_O] | 1 | 0.7% |
| Cd[(SO_4_)_4_] | 1 | 0.7% |

**Table S4.** Statistics analysis of Cd^2+^ solvation structures of CSO-3LiCl with a coordination distance ≤ 3 Å

| Solvation structures | Number | Percentage |
| --- | --- | --- |
| Cd[ClSO_4_(H_2_O)_4_] | 37 | 23.0% |
| Cd[(SO_4_)_2_(H_2_O)_4_] | 31 | 19.2% |
| Cd[SO_4_(H_2_O)_5_] | 20 | 12.4% |
| Cd[Cl_2_(H_2_O)_4_] | 16 | 9.9% |
| Cd[(SO_4_)_2_(H_2_O)_3_] | 14 | 8.7% |
| Cd[(SO_4_)_3_(H_2_O)_2_] | 10 | 6.2% |
| Cd[Cl(SO_4_)_2_(H_2_O)_2_] | 8 | 5.0% |
| Cd[Cl_2_SO_4_(H_2_O)_3_] | 7 | 4.4% |
| Cd[ClSO_4_(H_2_O)_3_] | 6 | 3.7% |

**Table S5.** Comparison of cyclic stability and discharge voltage with other aqueous AF systems

| Aqueous AF system | Anode current collector | Electrolyte | Cathode | Cyclic stability | Discharge voltage |
| --- | --- | --- | --- | --- | --- |
| Zn-based batteries | Cu@AOF^[7]^ | 3 M Zn(OTf)_2_ | Zn_0.5_VO_2_ | 60% (2,000, 1 A g^−1^) | ~0.7 V |
|  | CuAI^[8]^ | Zn(OTf)_2_/PAM hydrogel | Zn*_x_*VO_2_ | ~50% (300, 1 A g^−1^) | ~0.9 V |
|  | HCl-modified Cu foil^[9]^ | 2 M ZnSO_4_ + 0.2 M MnSO_4_ | Zn*_x_*MnO_2_ | 80% (500, 1 A g^−1^) | ~1.2 V |
|  | Cu foil^[10]^ | 1 M ZnSO_4_ + 4 M EMImCl | Zn*_x_*PANI | 78.8% (300, 1 A g^−1^) | ~1.0 V |
|  | Cu foil^[11]^ | 1 m Zn(OAc)_2_ + betaine | ZnI_2_ | 97.4% (1,000, 0.15 A g^−1^) | ~1.2 V |
|  | Cu foil^[12]^ | 4 m Zn(OTf)_2_ + 3 m TAA | Zn*_x_*a-V_2_O_5_@Gr | 71.4% (100, 10 A g^−1^) | ~0.7 V |
|  | Cu foil^[13]^ | 2 M ZnSO_4_ + 50 mM Mu | ZnI_2_ | 55% (1,000, 5 A g^−1^) | ~1.2 V |
|  | Cu foil^[14]^ | 2 M Zn(OTf)_2_ in 50 vol% PC | ZnMn_2_O_4_ | 80% (275, 0.35 A g^−1^) | ~1.2 V |
|  | Cu foil^[15]^ | 2 m Zn(OTf)_2_ in 70 vol% SL | Zn_0.25+_*_x_*V_2_O_5_ | 85% (100, 0.45 A g^−1^) | ~0.7 V |
|  | Ti foil^[16]^ | 4 m Zn(OTf)_2_ + 0.5 m Me_3_EtNOTf | Zn*_x_*VOPO_4_ | 80% (90, 0.5 mA cm^−2^) | ~1.0 V |
| Sn-based batteries | Cu foil^[17]^ | 1 M Sn(CH_3_SO_3_)_2_ + 3 M CH_3_SO_3_H\|\|3 M H_2_SO_4_ | TCBQ | 78% (1,000, 10 mA cm^−2^) | ~0.8 V |
|  | Cu foil^[18]^ | 1 M Sn(CH_3_SO_3_)_2_ + 3 M CH_3_SO_3_H + 0.1 M TU | TCBQ | 62% (570, 1 A g^−1^) | ~0.8 V |
| Cd-based batteries | Cu foil (This work) | CSO-3LiCl | Bu_4_NBr-CdBr_2_ | 87.6% (2,000, 4 C) | 1.45 V |

**Table S6.** Comparison of AU and energy density with reported Zn-based pouch cells

| Cathode type | Cathode | Electrolyte | AU (%) | Energy density (Wh kg^−1^)* |
| --- | --- | --- | --- | --- |
| Vanadium-based cathode | VOPO_4_·2H_2_O^[19]^ | ZLT-DMC | 43 | 100 |
|  | Zn_0.25_V_2_O_5_·nH_2_O^[20]^ | Sulfolane/H_2_O | 31.2 | 94 |
|  | NH_4_V_4_O_10_^[21]^ | PMS additive | 10 | 40.4 |
|  | NaV_3_O_8_·1.5H_2_O^[22]^ | Pyridine additive | 22.2 | 64 |
|  | Zn_0.25_V_2_O_5_·nH_2_O^[23]^ | 12-C-4 additive | 45 | 101 |
| Manganese-based cathode | Mg-MnO_2_^[24]^ | BDOL/MeOH additive | 28 | 121 |
|  |  |  | 41.7 | 130 |
|  | MnO_2_^[25]^ | MPVMT additive | 11 | 92 |
| Halogen-based cathode | I_2_^[26]^ | Silk protein additive | 20 | 80 |
|  | This work | CSO-3LiCl | 100 | 157 |

*Energy density is calculated based on the mass of active materials in the cathode and anode.

**References**

[1] J. C. Phillips, R. Braun, W. Wang, J. Gumbart, E. Tajkhorshid, E. Villa, C. Chipot, R. D. Skeel, L. Kalé, K. Schulten, *J. Comput. Chem.* **2005**, 26, 1781-1802, <https://doi.org/10.1002/jcc.20289>

[2] J. Huang, A. D. MacKerell Jr, *J. Comput. Chem.* **2013**, 34, 2135-2145, <https://doi.org/10.1002/jcc.23354>

[3] P. Mark, L. Nilsson, *J. Phys. Chem. A* **2001**, 105, 9954-9960, <https://doi.org/10.1021/jp003020w>

[4] S. E. Feller, Y. Zhang, R. W. Pastor, B. R. Brooks, *J. Chem. Phys* **1995**, 103, 4613-4621, <https://doi.org/10.1063/1.470648>

[5] S. NosÉ, *Mol. Phys.* **2002**, 100, 191-198, <https://doi.org/10.1080/00268970110089108>

[6] S. Trasatti, *J. Electroanal. Chem. Interfacial Electrochem.* **1972**, 39, 163-184, <https://doi.org/10.1016/S0022-0728(72)80485-6>

[7] C. Wang, D. Wang, D. Lv, H. Peng, X. Song, J. Yang, Y. Qian, *Adv. Energy Mater.* **2023**, 13, 2204388, <https://doi.org/10.1002/aenm.202204388>

[8] T. Wang, Y. Xiao, S. Tang, W. Xiang, J. S. Yu, *Adv. Energy Mater.* **2025**, 15, 2500430, <https://doi.org/10.1002/aenm.202500430>

[9] Y. Shen, Y. Jiao, C. Wang, J. Zou, P. Li, S. Chen, M. Zeng, L. Fu, *Adv. Funct. Mater.* **2025**, 35, 2504042, <https://doi.org/10.1002/adfm.202504042>

[10] Q. Zhang, Y. Ma, Y. Lu, X. Zhou, L. Lin, L. Li, Z. Yan, Q. Zhao, K. Zhang, J. Chen, *Angew. Chem. Int. Ed.* **2021**, 60, 23357-23364, <https://doi.org/10.1002/anie.202109682>

[11] J. Wang, B. Zhang, S. Luo, X. Huang, A. Duan, H. Chen, W. Sun, *Angew. Chem. Int. Ed.* **2025**, 64, e202510354, <https://doi.org/10.1002/anie.202510354>

[12] H.-I. Kim, K. M. Lee, W.-Y. Kim, S. H. Kweon, X. Wang, S. Zheng, S.-H. Kim, J. H. Ha, S. J. Kang, Z.-S. Wu, S. K. Kwak, S.-Y. Lee, *Energy Environ. Sci.* **2024**, 17, 1961-1974, <https://doi.org/10.1039/D3EE02535G>

[13] M. Wang, J. Ma, Y. Meng, P. Tong, R. Luo, D. Shen, X. Zheng, N. Chen, M. Zhang, L. Song, Z. Zhang, D. Li, C. Wang, H. Cheng, Y. Lu, Z. Li, W. Chen, *eScience* **2025**, 5, 100397, <https://doi.org/10.1016/j.esci.2025.100397>

[14] F. Ming, Y. Zhu, G. Huang, A.-H. Emwas, H. Liang, Y. Cui, H. N. Alshareef, *J. Am. Chem. Soc.* **2022**, 144, 7160-7170, <https://doi.org/10.1021/jacs.1c12764>

[15] C. Li, R. Kingsbury, A. S. Thind, A. Shyamsunder, T. T. Fister, R. F. Klie, K. A. Persson, L. F. Nazar, *Nat. Commun.* **2023**, 14, 3067, <https://doi.org/10.1038/s41467-023-38460-2>

[16] L. Cao, D. Li, T. Pollard, T. Deng, B. Zhang, C. Yang, L. Chen, J. Vatamanu, E. Hu, M. J. Hourwitz, L. Ma, M. Ding, Q. Li, S. Hou, K. Gaskell, J. T. Fourkas, X.-Q. Yang, K. Xu, O. Borodin, C. Wang, *Nat. Nanotechnol.* **2021**, 16, 902-910, <https://doi.org/10.1038/s41565-021-00905-4>

[17] Z. Yu, Q. Wang, Y. Li, F. Zhang, X. Ma, X. Zhang, Y. Wang, J. Huang, Y. Xia, *Joule* **2024**, 8, 1063-1079, <https://doi.org/10.1016/j.joule.2024.03.007>

[18] F. Zhang, X. Zhang, Y. Shu, H. Xiao, Q. Wang, Q. Guo, Y. Wang, J. Huang, Y. Xia, *Angew. Chem. Int. Ed.* **2025**, 64, e202425419, <https://doi.org/10.1002/anie.202425419>

[19] H. Jiang, L. Tang, Y. Fu, S. Wang, S. K. Sandstrom, A. M. Scida, G. Li, D. Hoang, J. J. Hong, N.-C. Chiu, K. C. Stylianou, W. F. Stickle, D. Wang, J. Li, P. A. Greaney, C. Fang, X. Ji, *Nat. Sustain.* **2023**, 6, 806-815, <https://doi.org/10.1038/s41893-023-01092-x>

[20] Y. Wang, T. Wang, S. Bu, J. Zhu, Y. Wang, R. Zhang, H. Hong, W. Zhang, J. Fan, C. Zhi, *Nat. Commun.* **2023**, 14, 1828, <https://doi.org/10.1038/s41467-023-37524-7>

[21] Y. Song, M. Chen, Z. Zhong, Z. Liu, S. Liang, G. Fang, *Nat. Commun.* **2025**, 16, 3142, <https://doi.org/10.1038/s41467-025-58153-2>

[22] J. Luo, L. Xu, Y. Zhou, T. Yan, Y. Shao, D. Yang, L. Zhang, Z. Xia, T. Wang, L. Zhang, T. Cheng, Y. Shao, *Angew. Chem. Int. Ed.* **2023**, 62, e202302302, <https://doi.org/10.1002/anie.202302302>

[23] Y. Wang, B. Liang, J. Zhu, G. Li, Q. Li, R. Ye, J. Fan, C. Zhi, *Angew. Chem. Int. Ed.* **2023**, 62, e202302583, <https://doi.org/10.1002/anie.202302583>

[24] J. Ji, H. Du, Z. Zhu, X. Qi, F. Zhou, R. Li, R. Jiang, L. Qie, Y. Huang, *Angew. Chem. Int. Ed.* **2025**, 64, e202414562, <https://doi.org/10.1002/anie.202414562>

[25] Z. Zheng, X. Zhong, Q. Zhang, M. Zhang, L. Dai, X. Xiao, J. Xu, M. Jiao, B. Wang, H. Li, Y. Jia, R. Mao, G. Zhou, *Nat. Commun.* **2024**, 15, 753, <https://doi.org/10.1038/s41467-024-44893-0>

[26] S.-J. Zhang, J. Hao, H. Wu, Q. Chen, C. Ye, S.-Z. Qiao, *Adv. Mater.* **2024**, 36, 2404011, <https://doi.org/10.1002/adma.202404011>
